# Optimizing the Calculation of Free Energy Differences in Nonequilibrium Work SQM/MM Switching Simulations

**DOI:** 10.1021/acs.jpcb.2c00696

**Published:** 2022-04-11

**Authors:** Andreas Schöller, Fiona Kearns, H. Lee Woodcock, Stefan Boresch

**Affiliations:** † Faculty of Chemistry, Department of Computational Biological Chemistry, University of Vienna, Währingerstrasse 17, A-1090 Vienna, Austria; ‡ Department of Chemistry, University of South Florida, 4202 E. Fowler Avenue, CHE205, Tampa, Florida 33620-5250, United States; § Vienna Doctoral School in Chemistry (DoSChem), University of Vienna, Währingerstrasse 42, A-1090 Vienna, Austria

## Abstract

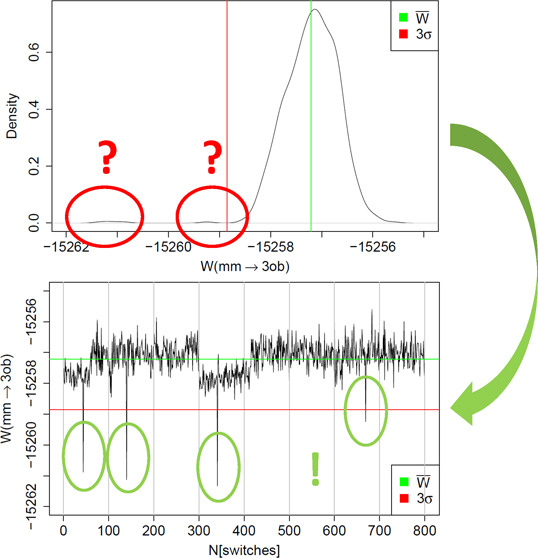

A key step during indirect alchemical free energy simulations using quantum mechanical/molecular mechanical (QM/MM) hybrid potential energy functions is the calculation of the free energy difference Δ*A*^low→high^ between the low level (e.g., pure MM) and the high level of theory (QM/MM). A reliable approach uses nonequilibrium work (NEW) switching simulations in combination with Jarzynski’s equation; however, it is computationally expensive. In this study, we investigate whether it is more efficient to use more shorter switches or fewer but longer switches. We compare results obtained with various protocols to reference free energy differences calculated with Crooks’ equation. The central finding is that fewer longer switches give better converged results. As few as 200 sufficiently long switches lead to Δ*A*^low→high^ values in good agreement with the reference results. This optimized protocol reduces the computational cost by a factor of 40 compared to earlier work. We also describe two tools/ways of analyzing the raw data to detect sources of poor convergence. Specifically, we find it helpful to analyze the raw data (work values from the NEW switching simulations) in a quasi-time series-like manner. Principal component analysis helps to detect cases where one or more conformational degrees of freedom are different at the low and high level of theory.

## Introduction

One of the major challenges in chemistry and biochemistry is elucidating thermodynamics and kinetics of enzyme–substrate interactions at the angstrom level. Force field-based methods, in particular molecular dynamics (MD) simulations, have become an essential method in this area. They yield coordinates and velocities of each atom in the system as a function of simulation time, and through statistical mechanics these raw data can be connected to macroscopic properties at microscopic resolution.^1,2^ One application of particular interest is the calculation of free energy differences (Δ*A*), the principal determinant of whether or not a chemical or biological process will proceed spontaneously.^3−5^ Areas in which free energy simulations are used routinely include the computation of relative binding affinities in drug development^6^ as well as the calculation of absolute binding affinities.^7^ These methods do not only provide predictions of relative or absolute binding free energy differences but help to better understand the mechanisms of protein–ligand^8,9^ as well as protein–protein interactions.^10−12^ Whenever a chemical process of interest requires electronic scale details that molecular mechanics (MM) force field cannot provide, the tool of choice is hybrid quantum mechanical/molecular mechanical (QM/MM) calculations^13−16^ for which the Nobel prize was awarded in 2013.^17^ The need for QM/MM methods arises in many circumstances, for example, prediction of p*K*_a_ values in complex environments, accurate description of metal ions, and many more.^18−21^ While free energy simulations (FES) with MM force fields has become routine, QM/MM calculations remain computationally demanding, which limits the applicability of QM/MM FES.^22,23^ Furthermore, several technical tricks routinely used in MM calculations of Δ*A* cease to work at the QM/MM level.^24^ Therefore, FES at the QM/MM level of theory at acceptable cost^25^ remains an elusive goal.

One common strategy to circumvent these problems is to employ an indirect thermodynamic cycle, which takes advantage of the inherent characteristic of free energy being a state function.^24^ Suppose we want to compute a (relative) binding free energy difference (Δ)Δ*A*_*X*→*Y*_ for two ligands *X* and *Y*. Instead of computing the required alchemical free energy differences Δ*A*_*X*→*Y*_^high^ directly at a high level of theory, for example, QM/MM, the alchemical transformation is carried out at a low(er) level of theory, for example, MM. The cycle is closed by the calculation of the free energy differences between the low and high level of theory at the end points. So in order to calculate Δ*A*_*X*→*Y*_^high^, the direct transformation Δ*A*_*X*→*Y*_^low^ and the two correction steps Δ*A*_*X*_^low→high^ and Δ*A*_*Y*_^low→high^ need to be computed. Although calculating the difficult to obtain quantity Δ*A*_*X*→*Y*_^high^ by means of three simpler and cheaper steps seems enticing, the accurate calculation of the correction steps is crucial and often a cause of failure due to convergence problems and the need for extensive conformational sampling.^26,27^ Further, the need for QM/MM energy/force evaluation during the correction steps makes them per se an expensive task.

Various approaches to compute the corrections Δ*A*^low→high^ reliably, sometimes referred to as “bookending” corrections, have been suggested.^28−31^ We showed in previous work that the use of nonequilibrium work simulations (NEW)^32^ leads to converged values for the correction free energies Δ*A*^low→high^ where other approaches fail.^33−35^ Since NEW calculations connecting two levels of theory can easily become computationally impractical, it is imperative to keep the cost of such calculations manageable to make real world applications feasible. A related challenge is to find reliable indicators whether a particular NEW switching protocol has resulted in converged results..^27^ In the context of classical mechanical interactions, a large body of work^36−39^ is concerned with optimizing calculations using Jarzinsky’s and Crooks’^40^ relations. Some studies have attempted to tune switching protocols in NEW simulations.^41−43^ For example, by varying how the coupling parameter, λ, changes during the alchemical FES (rate, as well as shape of the switching protocol), the average work could be minimized although the effect on statistical error and computational cost of the optimized protocols was less clear.^43^

In the present context, that is, when attempting to compute Δ*A*^low→high^, the most crucial factor is the computational cost of the force/energy evaluations during the switches since these require calculations at the high level of theory. The overall mitigating factor is that one needs multiple switches, which are completely independent calculations. In other words, the computational cost of NEW switching simulations is a trivially parallelizable task. This brings up the immediate question: is it more efficient to carry out a large number of short switches, or does one achieve better convergence of Δ*A*^low→high^ with fewer switches of longer simulation length? To formulate a concrete example, which protocol leads to the best converged and most accurate results: 10 000 switches of 100 MD steps each, 1000 switches of 1000 MD steps each, or 100 switches of 10 000 MD steps each? On a single processor, the computational cost of the three approaches is identical. If one has a large number of processors/machines available, the first protocol will be computationally the most efficient; however, it may not give the most accurate/precise result. For classical force fields, for example, Hummer showed early on that fewer, longer switching simulations lead to better convergence and, hence, more accurate results.^36^ Recently, Aldeghi et al. carried out an analysis into this question to optimize protocols for the calculation of absolute binding free energies when using classical force fields and suggested switching lengths of 80 ps.^44^ Such switching lengths, however, would be prohibitively costly when employing QM/MM Hamiltonians; ideally, we would want to carry out NEW switches of less than a few thousand MD steps. Since switching lengths over fractions of a picosecond or just a few picoseconds most likely result in end points far away from equilibrium, we wanted to ascertain that the findings of, for example, Hummer^36^ hold when computing Δ*A*^low→high^ using, by necessity, very short switching simulations. Therefore, finding a good balance between computational efficiency and accuracy of the results is the primary goal of this study.

In a recent work,^35^ we introduced the “HiPen” test set, a collection of 22 molecules for which we analyzed the convergence of Δ*A*^low→high^. The compounds were selected from the Maybridge Hitfinder library of drug-like compounds^45^ based on several criteria. In particular, we picked molecules based on the penalty score when assigning CGenFF 3.0 parameters.^46,47^ Parameter assignment by CGenFF (in particular dihedral angle parameters, partial charges) relies on molecular similarity. A high penalty score indicates that no close model compound could be found in CGenFF’s database; therefore, the resulting parameters should be understood as an “educated guess” only. In the present context, we expected that such “high penalty” force field parameters, at least for some of these compounds, would make it challenging to compute the correction Δ*A*^low→high^, even with NEW based switching methods. On the basis of the convergence and agreement with reference results, the compounds were classified as “good”, “bad,” or “ugly”. In ref (35), a single switching length was used based on previous experience with model compounds. Similarly, a large number of switches (10 000 per Δ*A*^low→high^) were carried out in an attempt to ensure convergence. Thus, our starting point is to see whether this protocol can be optimized, in particular whether comparable results can be achieved at significantly lower computational cost by varying switching length, number of switching simulations, or both. As in ref (35), we only compute Δ*A*^low→high^ for the individual test molecules in the gas phase.

When initially using the simulation protocols described in ref (35), we observed a few discrepancies to our earlier results. This prompted us to search for ways to discern and understand factors hindering convergence. In particular, we devised two approaches/tools which not only helped us resolve the deviations from the earlier results but which are generally useful to spot problems when attempting to compute free energy differences between two levels of theory. First, the raw data needed for Jarzynski’s equation^32^ are the work values of changing the Hamiltonian from the low to the high level of theory (cf. Theory and Methods). A priori, there is no temporal ordering in these work values. However, the switching simulations are started from restart files generated from equilibrium simulations at the low of level theory; thus, there is temporal ordering of how the starting points were generated. Plotting the work values as if they formed a time series helped us detect that in some cases our equilibration simulations had been too short.

Second, even after repeating the affected calculations with a longer equilibration, for some systems we still observed a (very) small number of switches with outlying work values (i.e., work values deviating significantly from the mean). When we analyzed convergence problems in earlier work,^33,34^ we always found strong indications that these were caused from different preferred conformational substates at the two levels of theory. Investigating such effects systematically quickly becomes difficult once multiple dihedral angles are involved. A possibly helpful tool is principal component analysis (PCA),^48^ widely used in multivariate data analysis, which not only detects correlations between multiple independent variables, but also displays the relative contribution to the variance for each variable (loadings) in a predictive way. Therefore, we explored whether PCA makes it possible to identify problematic conformational degrees of freedom, for example, dihedral angles, in a semiautomatic manner.

The remainder of the manuscript is organized as follows. In Theory and Methods, we first summarize the theoretical background and introduce the model systems used for validation. Next, we present the technical details of all simulations carried out. Then, we describe the quasi-time series approach to analyze work values from nonequilibrium work (NEW) simulations, as well as our use of principal component analysis (PCA). In Results, we begin by first showing data obtained based on the protocols from the original HiPen study^35^ and analyze cases where convergence was unexpectedly poor using the quasi-time series approach. This led to the use of extended equilibration simulations and the final optimized protocols. We conclude by presenting illustrative results obtained by PCA.

## Theory and Methods

### Theoretical Background

The focus of this study is the free energy difference between two levels of theory, as needed in indirect cycle QM/MM FES. As in our previous work,^33−35^ we chose MM as the low level, and the semi-empirical (SQM) SCC-DFTB method^49^ as implemented in CHARMM^50^ as the high level of theory. Our aim is to compute Δ*A*^MM→SQM^ as accurately as possible while keeping the computational cost low. To obtain these free energy differences, we employed NEW simulations, using primarily Jarzynski’s equation^32^
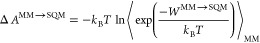
1In eq 1, *k*_B_ is Boltzmann’s constant, *T* is the temperature, and *W* is the work required to change the Hamiltonian from MM to SQM obtained from, as we call them, “switching simulations”, often only referred to as “switches”. As required by the theory behind eq 1, switches were started from restart files written at regular intervals during an equilibrium simulation at the MM level of theory at constant temperature and volume, hence the subscript MM. The averaging, indicated by the angular brackets ⟨⟩, was carried out over the work values obtained from *N*_replicate_ switches. This was one of the parameters we varied systematically with the other being *N*_switch_, that is, the number of MD steps used per switch (cf. below).

In ref (35), results calculated with eq 1 were not always converged. Thus, as in the previous work,^33−35^ we used Crooks’ equation^40^ to obtain reference results. The superiority of Crooks’ relation to reliably compute free energy differences when there is low overlap between distributions of forward and backward work values, that is, situations when Jarzinsky’s equation is expected to converge poorly, is well documented in the literature.^36−39^ To use Crooks’ relation, equilibrium simulations at the high level of theory, as well as switches from the high to the low level of theory (*W*^high→low^) are needed. Although this is too expensive for most practical applications, it is relatively affordable when using SQM as the high level of theory, permitting us to get reliable reference values for Δ*A*^MM→SQM^. Furthermore, several convergence criteria permitting one to estimate the quality of results obtained by Jarzynski’s equation require the knowledge of the distribution of forward and backward work values (see also Supporting Information (SI)).^27,35,37,51^

### Choice of Model Systems

We already described the HiPen test set in the Introduction. The classification of a molecule as “good”, “bad” or “ugly” in ref (35) meant that use of Jarzynski’s equation was sufficient to obtain converged results for Δ*A*^MM→SQM^ (“good”), was not sufficient, that is, only Crooks’ equation worked (“bad”), and even use of Crooks’ equation did not work (“ugly”). As it seemed futile to attempt optimizing calculations, where we encountered convergence problems even when using a very elaborate protocol, in this study we focused primarily on the “good” compounds from ref (35). Therefore, all “good” compounds plus one “bad” compound **21** from the full HiPen set were picked as the test pool for this study.

**Figure 1:**
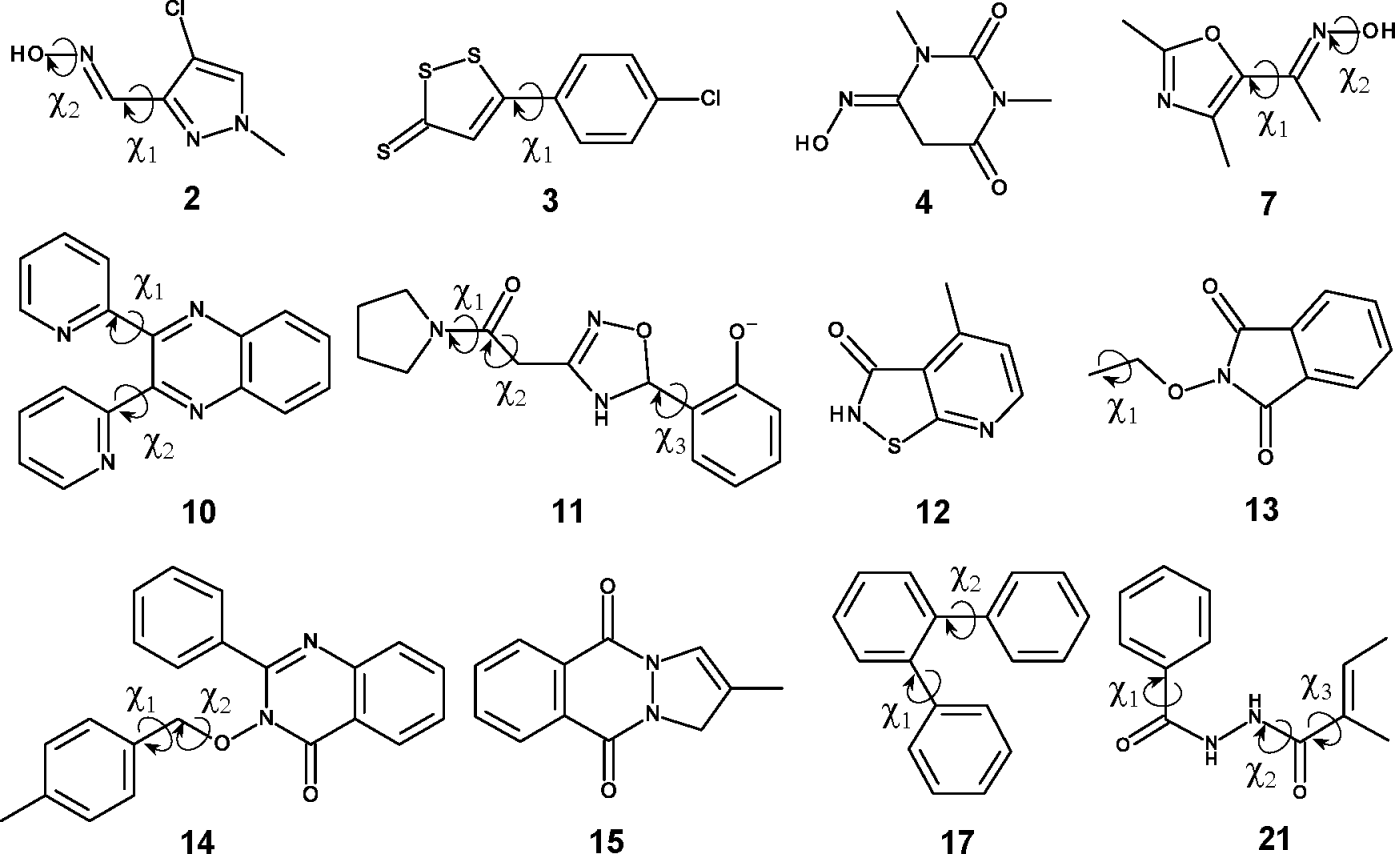
Subset of HiPen Data set considered in this work. Dihedral angles, which were candidates for randomization and analysis, are labeled.

These compounds are shown in Figure 1. To ease the comparison with ref (35), we keep the compound IDs used there. The figure also indicates and labels some dihedral angles which we considered to have an influence on the results. Where possible, the dihedral angle labels used in ref (35) were kept. Some additional details about the compounds, such as total number of atoms and number of heavy atoms, as well as the respective offset which was subtracted from all Δ*A*^low→high^ results, can be found in Table S1 of SI.

### Simulation Details

All simulations were carried out with CHARMM (developmental Version 44a2).^52^ All simulations were carried out in the absence of solvent, neither implicit, nor explicit (i.e., gas phase simulations).

### Generation of Starting Points for Nonequilibrium Switches

#### Preparation and Initial Equilibration

Geometry optimized starting coordinates, molecular topologies files and force field parameters were taken from the freely available repository of the HiPen Data set (DOI:10.5281/zenodo.2328952). For each molecule shown in Figure 1, eight starting coordinates were generated as follows: Rotatable bonds were randomized, then the coordinates were minimized for 1000 steps using the adopted basis set Newton–Raphson method^53,54^ while restraining the dihedral angles to the respective random values (harmonic dihedral restraints with a force constant of 100 kcal mol^–1^rad^–2^). After removing the restraints, Langevin dynamics (LD) was carried out to equilibrate the system at both the MM and SQM levels of theory. A friction coefficient of 5 ps^–1^ was applied to all atoms and random velocities were assigned at each step corresponding to a temperature bath of 300 K. Following the initial protocol from ref (35), the length of this equilibration simulation was 10 ps.

For five compounds, **2**, **7**, **10**, **12**, and **21**, all simulations described below were repeated using a more elaborate equilibration protocol. First, the length of the equilibration simulation was extended to 5 ns in the MM case and 500 ps in the SQM case. Second, for compound **7** the randomization of dihedral angles was modified compared to ref (35). In Figure S1, we show the dihedrals and their labels considered by Kearns et al. and compare them with the present work. As one can see, choosing χ_1_^old^ (Figure S1a) simply was an oversight; there is no point in randomizing a dihedral angle in a heterocycle. Similarly, randomizing χ_3_^old^ can lead to an isomerization around the C=N double bond (cis ↔ trans), which for oximes does not occur under the simulation conditions.^55^ Thus, we chose not to randomize these two dihedrals in the modified equilibration, prompting the change in labeling for the dihedrals (cf. Figure S1). For **7**, this left a single dihedral angle (i.e., χ_2_^old^ in the old; χ_1_ in the new labeling scheme) as a candidate for randomization. To understand the implications of randomizing χ_1_ (χ_2_^old^), we calculated potential energy scans at the MM and 3OB levels of theory (see Figure S2). The global minimum for dihedral χ_1_ is at ±180° with a secondary local minimum at 0°. Given that there are two minima, randomizing χ_1_ is an option. However, as the potential energy scans (see Figure S2) show, the barriers separating the two minima are high, and it seems unlikely that adequate sampling according to their Boltzmann weight would occur during the simulations. For this reason, we decided not to randomize χ_1_; its initial value was set to 180°. Note that the dihedral angle χ_2_ indicated in Figure 1 for compounds **2** and **7** was never considered for randomization; it is included in the figure because it turned out to be relevant for analysis.

#### MM Simulations

Eight LD simulations were carried out, starting from the eight coordinate sets obtained by the preparation and initial equilibration procedure just described. On top of the different coordinates, random initial velocities were assigned in each of the simulations. The simulation length was 10 ns, that is, 10 million LD steps with a time step of 1 fs. Restart files were written at every 1000th step. Thus, during a cumulative simulation length of 80 ns, 80 000 restart files were saved, serving as the pool to carry out nonequilibrium switching simulations to the high (SQM) level of theory. The molecules were fully flexible, and nonbonded interactions were not truncated (“infinite” cutoff radius).

#### SQM Simulations

For each of the eight starting structures equilibrated at the SQM level of theory, a LD simulation of 1 ns (1 million steps with a time step of 1 fs) was carried out. Restart files were written every 100th step, thus resulting in a total of 80 000 restart files generated during a cumulative simulation length of 8 ns. The self-consistent charge density functional tight-binding method as implemented in CHARMM^50^ with the 3ob-3-1 parameter set (https://www.dftb.org/parameters/download/3ob/3ob-3-1-cc/)^56−59^ was used.

#### Nonequilibrium Work Simulations

Using the restart files written during equilibrium simulations at the MM and SQM levels of theory, nonequilibrium switches MM → SQM and SQM → MM were carried out. As described in detail in earlier work,^33^ the mixing of Hamiltonians was done with the MSCALE functionality of CHARMM^60^ and the work value *W* during the switch was accumulated with CHARMM’s PERT module^52^ in slow-growth mode. The time step during the switching simulations was 1 fs. The Hamiltonian was changed linearly from MM to SQM (“forward”) and SQM to MM (“backward”) during a period of 200 fs (200 steps, NSWI200), 500 fs (500 steps, NSWI500), 1000 fs (1000 steps, NSWI1000) and 2000 fs (2000 steps, NSWI2000); cf. Table 1. A “reduced” protocol in which we used only a quarter of the switches generated for NSWI2000 is referred to as NSWI2000^red^.

**Table 1:** Combinations of Switching Length *N*_switch_ in fs and Number of Switches (*N*_replicate_) Studied^a^

	*N*_switch_ [fs]	*N*_replicate_	blocksize	*t*_total_ [ns]
NSWI200	200	8000	1000	1.6
NSWI500	500	3200	400	1.6
NSWI1000	1000	1600	200	1.6
NSWI2000	2000	800	100	1.6
NSWI2000^red^	2000	200	25	0.4

a Except for NSWI2000^red^, in which a reduced subset of the NSWI2000 data was used, the cumulative length of all switching steps *t*_total_ is identical in all cases to keep computational cost constant. The column “blocksize” indicates the number of switches started from restart files saved during each of the eight equilibrium simulations carried out for each system.

**Figure 2:**
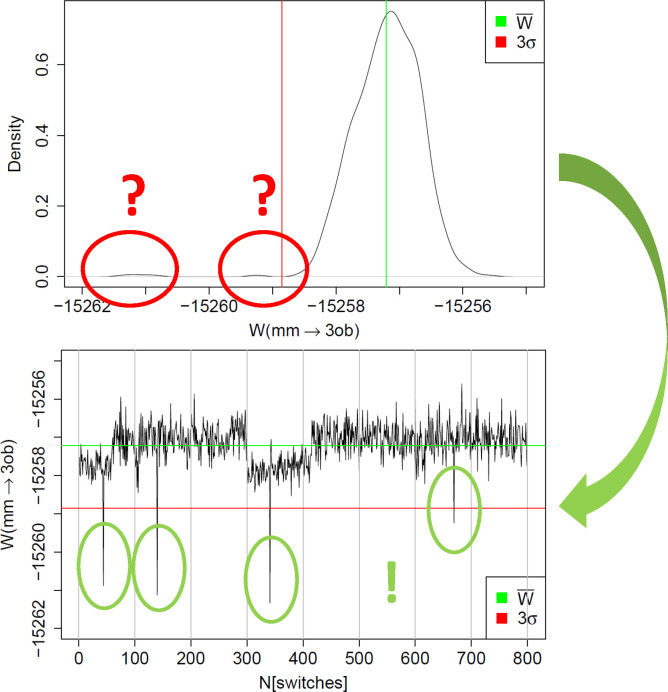
(Top) Example of a non-Gaussian distribution of MM → SQM work values *W*. Additional modes of more negative work values are circled in red. The average work value *W̅* is indicated by a green line and *W̅* – 3σ by a red line. (Bottom) Same data displayed as a “quasi” time series; green and red lines indicate *W̅* and *W̅* – 3σ as before. Individual switches with working values deviating by more than 3σ are highlighted by green circles. All work values are in kcal/mol.

Depending on the switching length, *N*_switch_, the nonequilibrium simulations were launched only from every 10th (NSWI200), 25th (NSWI500), 50th (NSWI1000), and 100th (NSWI2000) restart file saved during the equilibrium simulations. This results in the number of switches summarized in Table 1. Switches were carried out in both the forward and backward direction. While the focus of this work is on the forward (MM to SQM) direction using Jarzynski’s eq (eq 1), the backward work values were needed for histograms and densities of work distributions in the two directions, as well as to compute reference values using Crooks’ equation. The densities, as shown, for example, in Figure 2, were calculated with R (version 3.4.4)^61^ using the built-in density() function based on kernel density estimation without adjusting the bandwidth manually or giving any additional parameters (density.default).^62−66^

The major goal of this study is to search for an optimal combination of switching length *N*_switch_ and number of switches *N*_replicate_, or, phrased differently, to answer the question whether it is better to use many short switches or fewer longer switches. The combinations of *N*_switch_ and *N*_replicate_ listed in Table 1 make possible such a comparison as the cumulative number of simulation steps is identical for each of the four cases; that is, on a single processor the computational effort would be identical. In the following, the shorthands NSWI200, NSWI500, and so forth will not only be used to indicate switching lengths of 200 fs, 500 fs, and so forth, but to denote the respective protocols (combination of switching length and number of switches) shown in Table 1.

### “Quasi Time Series” Analysis

The distribution of work values obtained from forward and backward switches is often far from Gaussian; quite frequently it is even multimodal. A prototypical example is shown in the top plot of Figure 2; the data are for molecule **2** using the 10 ps equilibration simulation. The red circles indicate two clusters of work values which are noticeably more negative from the mean *W̅* (green line). The red line indicates *W̅* – 3σ; consequently, more negative work values are expected to occur rarely. Since highly negative work values have a significant weight in Jarzynski’s eq 1, they might be responsible for systematic deviations between results obtained by Jarzynski’s and Crooks’ equation, or be the reason for slow convergence.

From a distribution of work values, details about outliers leading to additional modes and/or shoulders in the main mode are difficult to discern. Ideally, the work values one uses are statistically independent. In the present work, the NSWI200 switches were started from restart files written in 10 ps intervals,and for the NSWI2000 switches the interval was 100 ps (cf. above). When computing the average in Jarzynski’s equation, the ordering of the work values is irrelevant. Nevertheless, the generation of the restart files introduces a partial temporal ordering. The eight simulations during which the restart files were written are indeed statistically independent (different initial velocities and coordinates). Within each of the simulations, however, restart files were written out in order and statistical independence cannot be guaranteed. We, therefore, decided to treat and plot our data (work values) as if they formed a time series.

This is shown in the bottom plot of Figure 2. The eight “sections” separated by thin gray lines correspond to the eight independent simulations during which restart files were written; we refer to these as “blocks”. Within such a section/block, the work values are plotted in the temporal order in which the restart files were saved. The example shown in the figure is for the NSWI2000 data, that is, 100 switches were started from the full set of restart files saved during the respective equilibrium simulation. Rather than resetting the counter between independent simulations, we number the switches from 1–100 for the first simulation, 101–200 for the second simulation, and so forth, as shown on the *x*-axis of the plot. We refer to this representation of the work values as a “quasi time series”. As in the density plot (top of Figure 2), *W̅* is indicated as a green and *W̅* – 3σ as a red line. Plotting the data in such a manner automatically leads to a simple labeling scheme for the individual switches; for example, switch 341 is the 41th NSWI2000 switch started from restart files saved during the fourth out of the eight independent equilibration simulations.

A plot as shown at the bottom of Figure 2 now makes it straightforward to pinpoint the switches leading to the effects reflected in the corresponding density plot. In particular, one can easily identify four work values (highlighted by green circles), which deviate from *W̅* by more than 3σ. In the following, we will often refer to work values with *W* < *W̅* – 3σ as “outliers”. The threshold *W̅* – 3σ is somewhat arbitrary, but it is an easily applied criterion to automatically detect and flag highly negative work values. The occurrence of such “outliers” does not indicate that something is wrong; given a sufficiently large number of NEW switches, such negative work values are to be expected. Nevertheless, it may be insightful to understand why a particular switch (switching path) results in a significantly more negative work value. In the quasi time series in Figure 2, one further sees two regions with work values somewhat more negative than *W̅*; these are responsible for the slight shoulder toward more negative work values in the main peak of the distribution of work values.

#### Principal Component Analysis (PCA)

To investigate the factors resulting in outliers, we used principal component analysis (PCA). As raw data, we used the work values and selected values of dihedral angles (cf. Figure 1) before (χ_*i*_^pre^) and after the switch (χ_*i*_^post^). PCA was carried out with R (version 3.4.4)^61^ using the built-in pcrcomp() function, which calculates the principal components via singular value decomposition, either on the unscaled or the scaled data matrix.^62,66,67^ Scaled PCA was employed for collective variables with different value-regimes (e.g., work values *W* and dihedral angles χ_*i*_); the unscaled version was used when including dihedral angles only. To gauge consistency, the cumulative sum of the principal components’ variance quantity was always calculated. Generating the input for the PCA is trivial and the computational effort is negligible. The work values are available anyways, and the CHARMM input scripts for carrying out the switching simulations were modified to save selected dihedrals before and after the nonequilibrium molecular dynamics run.

## Results

### Optimization of Switching Simulations

**Figure 3:**
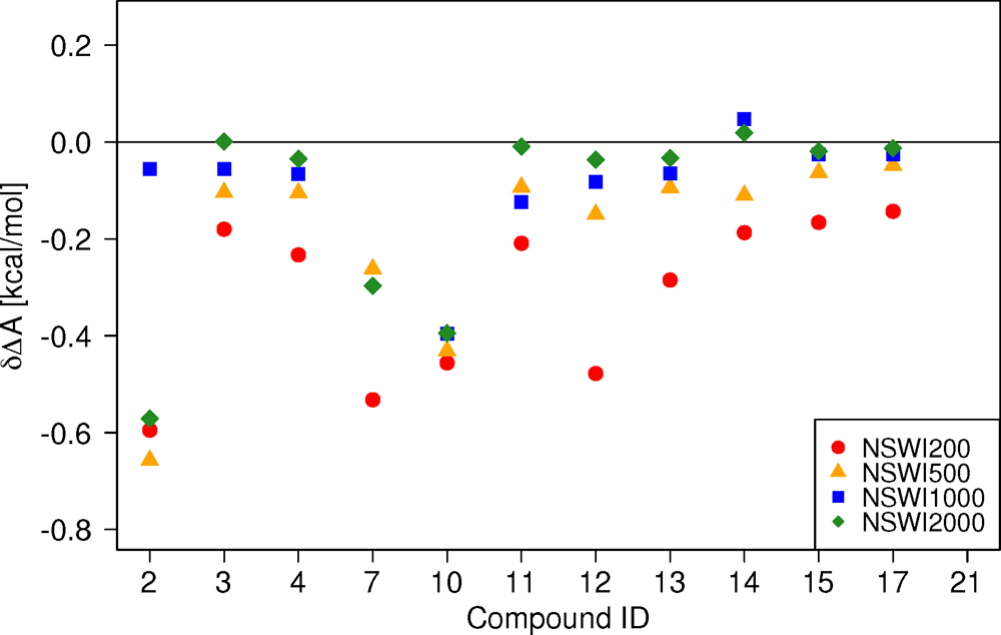
δΔ*A* (eq 2) for the results of the forward switching simulations (MM → SQM) using the original equilibration protocol as a function of switching length. Some values are off-scale; Figure S3 shows the same data including all values.

Figure 3 shows the results of the NEW-switching simulations in the forward (MM → SQM) direction obtained with the original short equilibration protocol. Each data point represents the difference
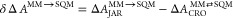
2between the free energy difference obtained by Jarzynski’s equation Δ*A*_JAR_^MM→SQM^ for a particular switching length and the best reference result obtained by Crooks’ equation Δ*A*_CRO_^MM⇄SQM^. Figure S4 is identical to Figure 3 but also includes error estimates. For detailed results of all conducted switching lengths, including convergence metrics, see Tables S3–S6. These tables also list the results obtained by Crooks’ relation for the switching protocols of different length; as one can see, there is almost no variation with switching length, indicating that these results are well converged. Some results are not included in Figure 3 as they are off-scale (specifically the NSWI1000 result for **7** (δΔ*A* = −1.6 kcal/mol, and all results for **21** (δΔ*A* = −6.1, −4.6, −3.3, and −1.0 kcal/mol for NSWI200, NSWI500, NSWI1000, and NSWI2000, respectively); these will be discussed shortly.

One can clearly see that in most cases the longest switching protocol (NSWI2000, green diamond) is closest to the respective reference result. Illustrative examples are, for example, **3** and **4**, where in terms of δΔ*A*, NSWI2000 < NSWI1000 < NSWI500 < NSWI200; that is, using 800 switches of 2000 fs leads to better results than 1600 switches of 1000 fs and so forth. The ordering is not always perfect; for example, for **11** the NSWI500 result is slightly better than NSWI1000; nevertheless, the lowest-magnitude δΔ*A* is obtained with the NWSI2000 protocol. While there are exceptions (**2**, **7**, **10**, and **21**), which will be analyzed next, Figure 3 strongly suggests that it is more efficient to conduct fewer longer switches than many short ones. In most cases, 800 2 ps switches delivered a more reliable result than 8000 200 fs switches while requiring the same computational effort.

**Figure 4:**
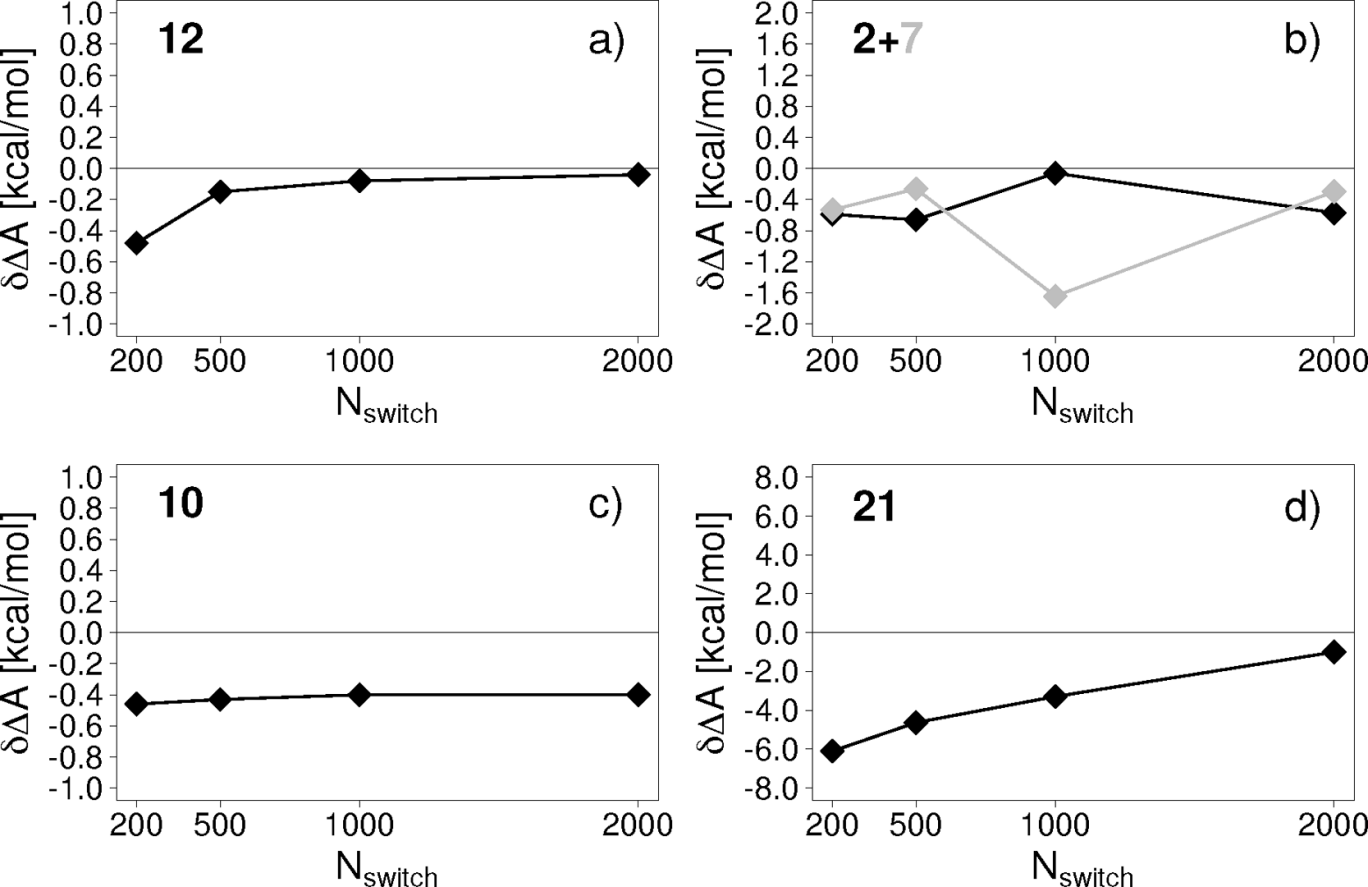
Detailed dependence of δΔ*A* on switching length for (a) compounds **12** (b) **2** and **7**, (c) **10**, and (d) **21**.

To better understand the problematic cases, we present more detailed plots of δΔ*A* as a function of switching length. In Figure 4, we plot δΔ*A* versus *N*_switch_ for compound **12**, our reference which has ideal behavior, as well as for three of the four outliers **2**, **10**, and **21**. These represent different “types” of deviation. The fourth outlier, **7**, behaved somewhat similarly to **2**. Both **2** (Figure 4b) and **7** do not obey the general trend that increasing switching length reduces δΔ*A*. For **2**, NSWI1000 gives an almost perfect result whereas the longer NSWI2000 protocol deviates from the reference result by almost as much as NSWI200 and NSWI500. In the case of **7**, NSWI500 and NSWI2000 perform well, but NSWI1000 deviates significantly from the reference result. Compound **10** exhibits the curious behavior of a systematic offset compared to the reference result, which does not become noticeably smaller as the switching length increases and remains at 0.4 kcal/mol even for NSWI2000 (Figure 4c). Finally, at first glance **21** behaves as expected (Figure 4d); that is, δΔ*A* becomes smaller as the switching length increases, but even when using switching lengths of 2 ps, the deviation to the reference result remains unacceptably high (∼−1 kcal/mol). In principle, the result for **21** was not surprising as it was classified as a “bad” compound.

### ”Quasi Time Series” Analysis of Switching Simulations

Our starting point to understand why the results for compounds **2**, **7**, **10**, and **21** failed to improve when increasing *N*_switch_ was to scrutinize their forward and backward work distributions. For all of them, the distributions were far from Gaussian, and in all cases there was a non-negligible number of switches with distinctively more negative work values. An illustrative example is shown in Figure 2. Since these outliers have high weight in Jarzynski’s equation, these were likely responsible for the systematic deviations of the forward results from the reference values obtained using Crooks’ equation.

**Figure 5:**
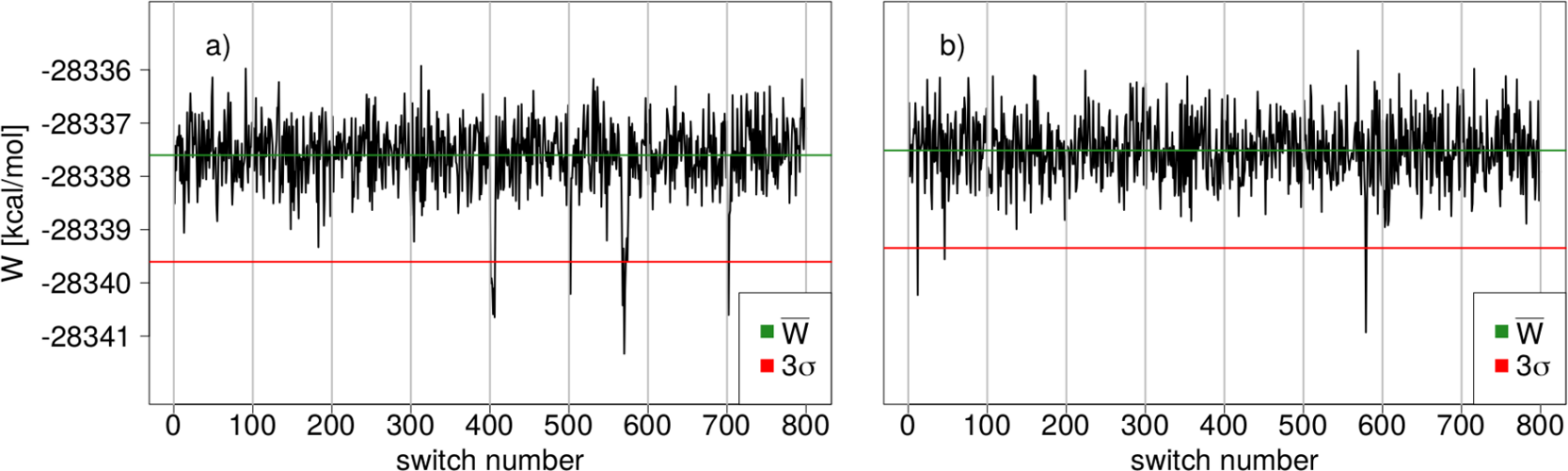
Quasi time series of Compound **10** plotted as *W* [kcal/mol] for the 2000 fs switches versus switch index, concatenating the underlying equilibrium simulations for (a) the original protocol and (b) for the modified protocol.

**Figure 6:**
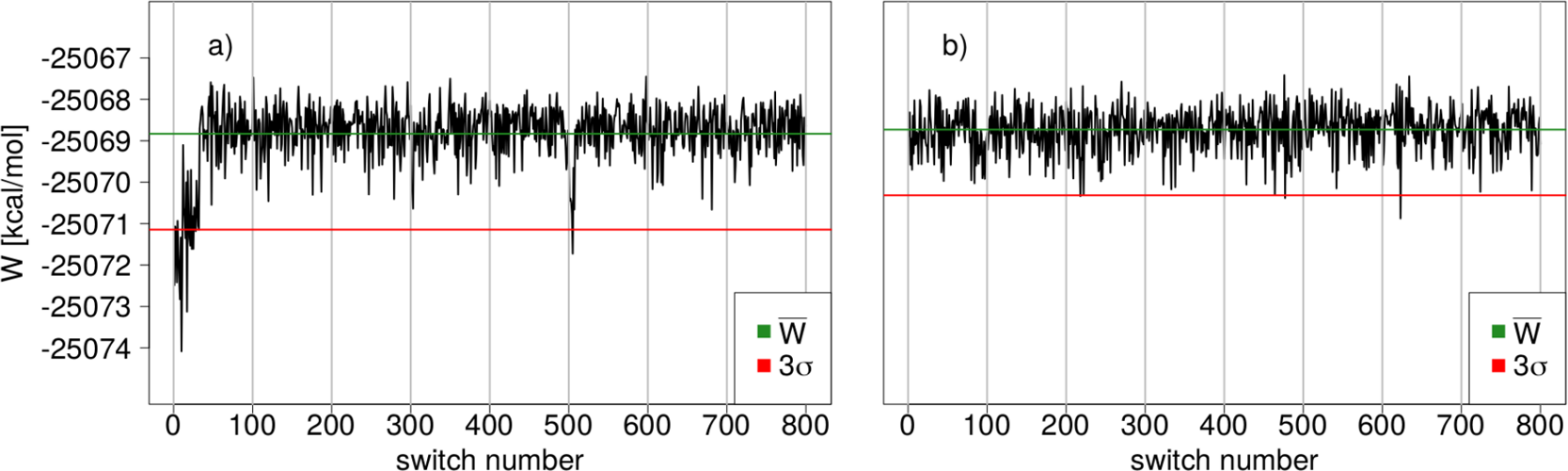
Quasi time series of Compound **21** plotted as *W* [kcal/mol] for the 2000 fs switches versus switch index, concatenating the underlying equilibrium simulations for (a) the original protocol and for (b) the modified protocol.

An obvious question to ask is whether these outliers occur at random, or whether there is some correlation to the order in which the starting coordinates (restart files) for the switches were generated. Therefore, as described in the subsection “Quasi Time Series” Analysis, we plotted the work values as a quasi time series. Illustrative examples for the initial simulation protocols (*N*_switch_ = 2000) are shown in Figure 5a for **10** and Figure 6a for **21**. The eight blocks of data (restart files) are marked by the vertical gray lines.

Focusing on negative outliers, work values *W*_*i*_ < *W̅* – 3σ_W_ (see the −3σ line in red in Figures 5 and 6), one notices that they occur often at the beginning of a replica, that is, at the beginning of one of the eight independent sets of starting points. For example, for **10** there are outliers near switch number 400, 500, and 700 (see Figure 5a), and for **21** there is a sizable number of switches with significantly more negative work values starting at switching number 0, as well as few values near switching number 500 (see Figure 6a). In other words, several switches started from restart files generated early during the respective equilibrium simulation at the MM level of theory led to unusually negative work values. Note that this is not always the case, for example, for **10** several switches after switch number 560 also give low work values (see Figure 5a). This observation prompted us to scrutinize our protocol for system preparation and equilibration (cf. Generation of Starting Points for Nonequilibrium Switches), and we repeated the full sequence of simulations for **2**, **7**, **10**, and **21** with the (much) longer equilibration simulations following the random dihedral angle assignment. To ensure that this did not affect the unproblematic cases, the longer protocol was also applied to **12**.

**Figure 7:**
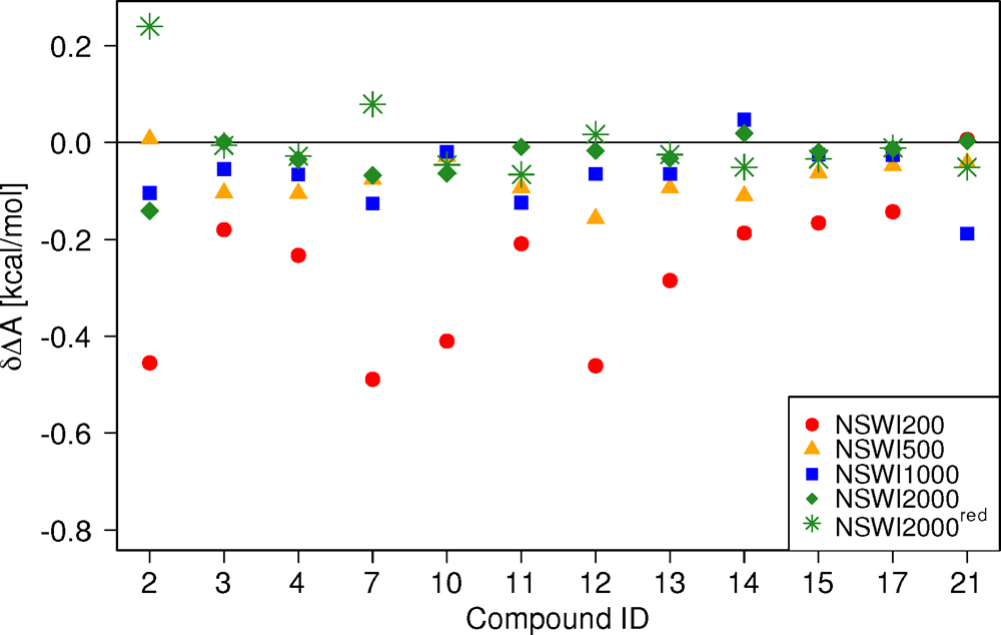
δΔ*A* calculated as the difference between two-sided method CRO and one-sided method JAR for switching forward (MM → SQM) after correcting the equilibration period for **2**, **7**, **10**, **12**, and **21** from 10 ps to 5 ns for MM and from 10 ps to 0.5 ns for SQM after random dihedral assignment procedure.

The effect on the quasi time series of the work values is displayed for **10** and **21** in Figures 5b and 6b, respectively. For **21**, all negative outliers have effectively disappeared, and for **10** their occurrence is much rarer. While we do not show the plots for **2** and **7** (see Figures S6 and S7), the overall picture is very similar. Occasional negative outliers, work values with *W*_*i*_ < *W̅* – 3σ_W_, remain, but their frequency is reduced. The modified protocol has a dramatic quality of the overall results, as can be seen in Figure 7 and Table 2. Now, the longest switching protocol NSWI2000 leads to the lowest deviation δΔ*A* from the reference in almost all cases; any deviations, such as for **7**, where δΔ*A*(NSWI1000) < δΔ*A*(NSWI2000), are so small as to be irrelevant in practice. Most surprisingly, **21** for which we had obtained large δΔ*A* values for all switching lengths, in line with its initial classification as “bad”, is now “perfectly behaved”. Full results for all compounds repeated with the longer equilibration protocol can be found in Tables S3–S6, and Figure S5 shows the data in Figure 7 with error bars. Figure S8 displays the convergence behavior as a function of switching length, analogously to Figure 4 above, for **12**, **2**, **10**, and **21**.

**Table 2:** MAD and Root Mean Square Error (RMSE) of Results Obtained from Forward Switches and Jarzynski’s Equation Compared to Reference Result Obtained by Crooks’ Equation (CRO) Including Corrected Results for **2**, **7**, **10**, and **21**

	MAD [kcal/mol]	RMSE [kcal/mol]
NSWI200	0.27	0.31
NSWI500	0.08	0.09
NSWI1000	0.08	0.09
NSWI2000	0.04	0.05
NSWI2000^red^	0.06	0.08

Having identified the NSWI2000 protocol as the most reliable method to compute free energy differences Δ*A*^low→high^ using Jarzynski’s equation, we attempted to reduce the computational effort further. Specifically, we lowered the number of switches per block from 100 to 25, that is, using *N*_replicate_ = 200 instead of 800. These results are labeled NSWI2000^red^ and are included in Figure 7 (green stars) and Table 2. While the mean absolute deviation (MAD) of NSWI2000^red^ is slightly larger than for NSWI2000, it is still smaller than MAD(NSWI1000) at one-quarter of the computational cost. As can be seen in Figure 7, most δΔ*A* values remain almost unchanged though, for example, for **2** there is already some deterioration.

The use of quasi time series analysis suggested corrections to our simulation protocol and dramatically improved the convergence of the computed Δ*A*^MM→SQM^ values. Nevertheless, as one sees, for example, in Figure 5b, even when employing the longer equilibration protocol some work values remain outliers, based on our criterion of deviations by more than three standard deviations from *W̅*. In principle, considerably more negative work values could bias the free energy difference computed by Jarzynski’s equation. To check whether this is the case here, we recomputed Δ*A*^low→high^ using Jarzynski’s equation for **2**, **7**, **10**, and **21**. The results for the work values from the NSWI2000 and NSWI2000^red^ switching protocols with the outliers present or removed are shown in Table 3.

**Table 3:** Outlier Impact on JAR (fw) for **2**, **7**, **10**, and **21** for NSWI2000 and NSWI2000^red^ with and without Outliers^a^

	*N*_Outliers_		δΔ*A*			
	NSWI2000	NSWI2000^red^	NSWI2000	NSWI2000*	NSWI2000^red^	NSWI2000^red^*
**2**	4	0	–0.14	0.26	0.24	0.24
**7**	4	1	–0.07	0.10	0.08	0.10
**10**	4	1	–0.06	0.12	–0.06	0.09
**21**	5	1	0.01	0.04	–0.05	0.01

a The results are displayed as δΔ*A* in kcal/mol, similarly to Figure 3 and Figure 7. *N*_Outliers_ is the number of outliers identified in the time series like analysis in the NSWI2000 and NSWI2000^red^ raw data. A * denotes results obtained with all outliers excluded from the calculation.

For **7**, **10**, and **21**, the difference in δΔ*A* with and without the outliers is less than 0.2 kcal/mol, which is negligible in practice. The free energy difference is shifted slightly to more positive values, as expected. For each of these three compounds, the reduced set of work values NSWI2000^red^ still contains one of the outliers. For **2**, the difference between the NSWI2000 (outliers included) and NSWI2000* (outliers excluded) results is 0.4 kcal/mol. While not negligible anymore, this is most likely still acceptable. More importantly, however, for **2** the reduced NSWI2000^red^ set does not contain a single outlier. As seen in Figure 7, **2** is the single case where using just 200 work values affected the result noticeably; this is the result of the reduced data set incidentally not containing any of the “outliers”.

### PCA of Switching Simulations

In earlier work,^34,35^ we showed that different conformational preferences at the two levels of theory had a noticeable impact on convergence. Therefore, it seemed likely that the outliers in terms of more negative work values were somehow correlated with dihedral degrees of freedom. We used PCA to investigate this question systematically and to identify the specific degree(s) of freedom responsible. As described in Principal Component Analysis, our input variables are the dihedral angles considered relevant before and after the switching simulations (see Figure 1), as well as the work values themselves. In the following, we focus on compounds **2** and **7**. For both systems, even using the extended equilibration protocol, we obtained isolated outliers in the work values (deviating by more than 3σ from *W̅*).

**Figure 8:**
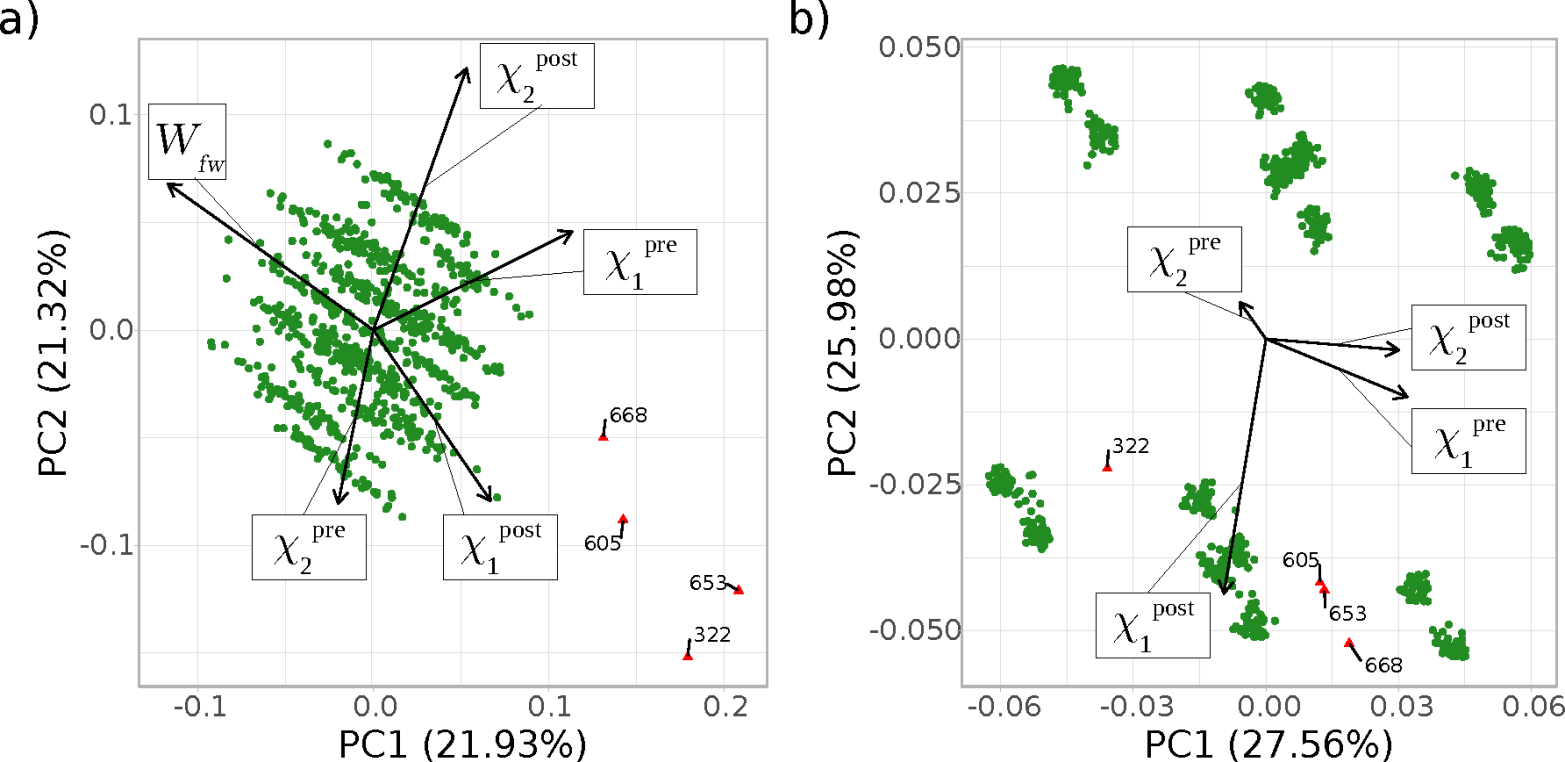
PCA of compound **2** plotted as PC1 versus PC2 for the longer equilibration protocol as (a) scaled version and (b) unscaled version. Outliers are labeled explicitly and marked by red triangles (rather than green dots) according to the predefined outlier criteria (deviations less than *W̅* – 3σ for panel a, and |χ̅_2_^post^| – 3σ for panel b, cf. “Quasi Time Series” Analysis).

Results of the PCA analysis for **2** are summarized in Figure 8. The data shown are for the final, long equilibration protocol; the plots for the original protocol can be found Figure S9. The corresponding quasi time series analysis is shown in Figure S6. Figure 8a is the scaled PCA in which dihedral values χ_1_, χ_2_, and work *W* were used as inputs. For the dihedral angles, we distinguish the values before (denoted as χ^pre^) and after (χ^post^) the switch. Highlighting of outliers, colored in red rather than green, and with explicit labels added, was done in an automated manner during the calculation/plotting of the PCA results according to the predefined criteria with respect to deviation from *W̅* or |χ̅_2_^post^|; cf. “Quasi Time Series” Analysis. The arrows in the plot are the loading vectors describing which feature of the input data correlates with the calculated principal component and in which way (positive/negative).

We start with the scaled PCA plot for **2** shown in Figure 8a. Four switches (322, 605, 653, and 668) behave very differently; based on outlier criterion *W̅*–3σ, they were colored in red automatically. These are exactly the switches which can be easily discerned in the quasi time series analysis; see Figure S6b. The four switches are located in the opposite direction of the loading vector for *W* indicating an unusual negative deviation of the feature *W*. Besides *W*, χ_2_^post^ has the largest loadings vector among all the variables, suggesting that this conformational degree of freedom may be of relevance as it accounts for a large proportion of the variance. In the unscaled PCA, shown in Figure 8b, we used |χ̅_2_^post^| – 3σ as the criterion for outlier detection (cf. “Quasi Time Series” Analysis). The only switches highlighted by the automated coloring are 322, 605, 653, and 668. This demonstrates that the four negative values in *W* are correlated and, possibly caused, by their χ_2_^post^ value.

Practically all switches from MM to SQM result in a χ_2_^post^ values around ±180° (green dots), meaning that the dihedral angle about the N–O bond is in the trans configuration. By contrast, the four outliers (colored in red) have χ_2_^post^ values of approximately 0°. In other words, these switches end in a cis configuration around the N–O bond. Thus, for compound **2** PCA not only detects the outliers but immediately makes clear how these switches differ from the rest.

**Figure 9:**
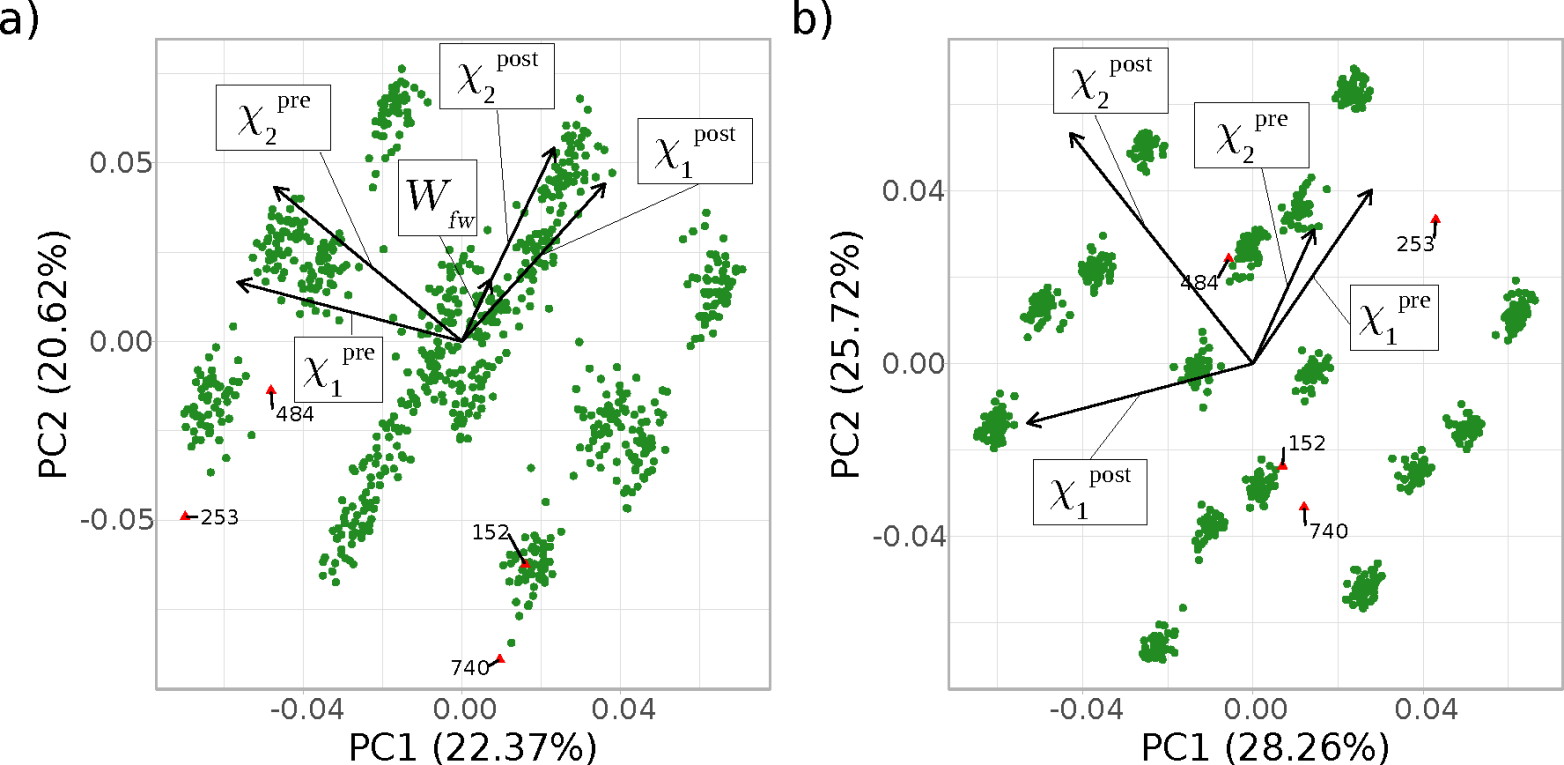
PCA of compound **7** plotted as PC1 versus PC2 for the longer equilibration protocol as (a) scaled version and (b) unscaled version. Outliers are labeled explicitly and marked by red triangles (rather than green dots) according to the predefined outlier criteria (deviations less than *W̅* – 3σ for panel a, and |χ̅_2_^post^| – 3σ for panel b, cf. “Quasi Time Series” Analysis).

Plots for the PCA of compound **7** when using the modified protocol are shown in Figure 9. The corresponding quasi time series and the PCA for the switches obtained with the original protocol can be found in Figures S7 and S10. Some similarities to **2** are expected as both molecules have an oxime moiety in close proximity to a hydrogen bond acceptor. Figure 9a shows the plot of the first two principal components for the scaled PCA. Labeling and coloring in red switches based on the *W̅* – 3σ criterion identifies the four outliers which can also be discerned in the quasi time series plot (Figure S7b). As for **2**, these four switches are located in the opposite direction of the loading vector for *W*. In this case, the loading vector **W** has the smallest magnitude of all the variables, thus contributing the least to the variance. On the other hand, the correlation with χ_2_^post^ is even more obvious because the corresponding loading vector points almost in the same direction as for *W*, indicating that both variables contribute in the same way to the variance (both variables contribute positively to PC1 and PC2). However, the magnitudes of the loading vectors for χ_1_, both pre and post, as well as χ_2_^pre^ are quite similar, indicating that all angles contribute equally to the variance. Two of the outliers, which were colored automatically in red, are reasonably well separated (253 and 740), whereas 152 is located in a cluster of “normal” switches and 484 is in close proximity to one. Studying the quasi time series (Figure S7b), one sees that 152 barely triggers the outlier criterion, which may explain the overlap with other switches. Overall, however, in the case of **7** the outliers are not as well separated as for **2**; the ability to color them automatically is needed.

Figure 9b shows the first two principle components of the unscaled PCA. Applying the |χ̅_2_^post^| – 3σ criterion identifies all four switches already discerned in Figure 9a. Analogous to what was observed for **2**, all switches start with χ_2_^pre^ around ±180°, and the four switches ending with χ_2_^post^ values around 0° result in unusually negative work values. While the outliers (with respect to χ_2_^post^) are somewhat separated from most of the other switches, they would be difficult to discern without the automatic coloring. Nevertheless, PCA facilitated the task considerably. In the scaled PCA, outliers are flagged, that is, colored in red, based on the *W* < *W̅* – 3σ criterion, and consideration of the loading vectors strongly indicated to analyze the role of χ_2_^post^. Moreover, it is straightforward to exclude other possibilities by applying the |χ| < |χ̅| – 3σ criterion to the remaining dihedral degrees of freedom. If one does this, very different switches with work values close to *W̅* are flagged. This shows that these degrees of freedom are not related to the occurrence of a highly negative work value. Since we use PCA as implemented in R (cf. Theory and Methods), these analyses can be carried out interactively and can be automated, at least to some degree. In this manner, such an analysis can be carried out in a short amount of time, even if more than two dihedral degrees of freedom need to be considered.

The analysis of both **2** and **7** indicates a strong correlation between the dihedral angle about the N–O bond after the switch and negative work values (*W* < *W̅* – 3σ_*W*_). As pointed out in earlier work,^34,35^ even when using NEW methods configurational mismatches between the different levels of theory are a possible reason for convergence problems. While in this specific case the convergence of the result was not affected significantly (see Table 3), the PCA analysis suggests that there are different conformational preferences at the low and high level of theory. A more detailed analysis of configurational sampling about χ_2_ at the two levels of theory, that is, of the production simulations during which restart files were generated, shows that at the MM level of theory the cis-configuration about the N–O bond of the oxime moiety present in **2** and **7** is never sampled. By contrast, at the SCC-DFTB level of theory values of |χ_2_^pre^| ≤ 53° were observed ∼13% of the time.

The rare forward switches, during which a trans → cis configurational change for χ_2_ occurs, lead to untypically negative work values. As previously stated, χ_2_ values around 0° are sampled at the SQM level of theory; therefore, such conformational changes during the switch are not artifacts. The remaining question is why they result in more negative work values. In Figure S2, we show a potential energy scan as a function of the χ_1_ and χ_2_ angles for **7** at the MM level of theory (λ = 0), the midpoint between MM and SQM (λ = 0.5), and the SQM level of theory (λ = 1). At the SQM end point, χ_2_ values of ±180° and 0° are quite close energetically, although χ_2_ = ±180° is the global minimum conformation. The comparison of the three energy surfaces shown in Figure S2 suggests the following explanation: As the potential energy surface changes from MM to SQM, a shallow minimum around χ_1_ = ±180°/χ_2_ = 0° develops, which is already clearly discernible in the plot for λ = 0.5 (Figure S2, middle). At λ = 0.5 the barrier separating the minima at χ_1_ = ±180°/±χ_2_ = 180° and χ_1_ = ±180°/χ_2_ = 0° is lower by about 2 kcal/mol than at the SQM end point (≈13% ≈ 4 instead of >6 kcal/mol). Therefore, conformational changes from χ_2_ = ±180° to 0° can certainly occur, although given the short switching length of 2 ps it is not surprising that they are rare. As one sees from Figure S2 (left), at the MM level of theory the minimum energy basin about χ_1_/χ_2_ = ±180°/±180° is quite wide, particularly in the χ_2_ direction. Thus, χ_2_ values of ±100° or even ±90° are quite accessible and are sampled at the MM level of theory. In other words, starting configurations |χ_2_| < 100° are close to the top of the barrier at λ = 0.5, and if their starting velocities point in the right direction the barrier can be overcome. Indeed, all four switches that ended in the χ_1_ = ±180°/χ_2_ = 0° minimum started from such configurations. As the switch progresses, the system then slides down toward the local energy minimum at χ_2_ = 0° at the SQM end point, resulting in a more negative work value compared to systems which remain around χ_1_/χ_2_ = ±180°/±180° throughout the switching simulation.

## Discussion/Conclusions

We systematically investigated the convergence of Jarzynski’s equation applied to computing Δ*A*^MM→SQM^ as a function of switching length and number of nonequilibrium work values used for averaging. The data clearly indicate that using fewer but longer switches leads to better converged and hence more accurate results. This is in line with earlier results,^36^ but it was important to ascertain that this does hold even in the regime of very short switching lengths, given that all the protocols which are computationally feasible, including NSWI2000, are far from equilibrium. Our findings lead to immediate practical benefits. Using the best protocol (800 switches of 2 ps length, NSWI2000) reduces the computational cost compared to the naive protocol of the original “HiPen” study^35^ by almost a factor of 10. Having identified the NSWI2000 protocol as a reliable way to compute Δ*A*^low→high^, we optimized it even further, 200 rather than 800 switches (NSWI2000^red^). With this final protocol, we obtained as good if not better results compared to ref (35) at 1/40 of the cost. At the same time, the present protocol remains trivially parallel, and if multiple computers and/or CPU cores are available one can compute the corrections Δ*A*^MM→SQM^ efficiently.

When attempting to understand possible sources of poor or slow convergence, two tools proved very helpful: (i) plotting the work values not only as a histogram but as a quasi time series, and (ii) employing PCA to detect correlations between outliers with respect to work values and conformational degrees of freedom before and after the switch. It should be stressed that these tools/utilities require as input either quantities one needs to compute anyways, such as the nonequilibrium work values, or quantities which can be calculated extremely fast, such as dihedral angles. In other words, very useful insights can be obtained at little or no cost. We employed both methods using the statistical software system R, which provides a convenient interface for plotting and carrying out the PCA. This facilitated detecting and understanding the source of outliers considerably. However, once the raw data are available, various programs could be used for these analyses.

In all cases which we analyzed in detail, the cause of outlying work values could be traced to a conformational degree of freedom, which for these relatively simple systems was always a dihedral angle. In many applications of computational chemistry, rotamers need to be enumerated and/or the corresponding dihedral angles identified. Our experience suggests that such degrees of freedom should be routinely analyzed, for example, by PCA, to detect and understand possible convergence problems.

In this study we deliberately focused on the “good” compounds from the full “HiPen” data set. More efficient protocols to obtain the work values needed for Jarzynski’s equation may not be enough for the “bad” and “ugly” cases. Here, techniques like force matching^68^ to make the low level of theory more high level like or the judicious use of intermediate stages to carry out the low to high transformation^31^ may be needed. Further, better equilibration and sampling strategies, such as self-guided Langevin dynamics (SGLD)^69,70^ in preparation for the equilibrium simulations during which the starting points for the switching step are written, should prove helpful. The combination with optimized protocols to obtain the nonequilibrium work values needed will make it possible to compute the correction Δ*A*^MM→SQM^ reliably and with sufficient efficiency.

## Supporting Information Available

Table S1, additional details of model compounds; Table S2, summary statistics for simulations with the short equilibration protocol; Tables S3–S6, detailed results for data plotted in Figures 3 and 7, as well as Figures S3–S5; Figure S1, change in dihedral angle labeling for compound **7**; Figure S2, potential energy scan about dihedral angles χ_1_ and χ_2_ for **7**; Figures S3–S5, alternativ plots of results; Figures S6 and S7, quasi time series analysis for **2** and **7**; Figure S8, convergence of the results as a function of the switching length for **12**, **2**, **7**, **10**, and **21** when using the extended equilibration protocol; Figures S9 and S10, PCA of data for **2** and **7** generated with the initial, short equilibration protocol (PDF)

## Abbreviations

MD: Molecular dynamics
MM: Molecular mechanics
(S)QM: (Semiempirical-)quantum mechanics
(S)QM/MM: (Semiempirical-)quantum mechanical/molecular mechanical hybrid methods
FES: Free energy simulation
NEW: Nonequilibrium work methods
JAR: Jarzynski’s Equation
CRO: Crooks’ Equation
