# Supplementary material for: Optimizing the Calculation of Free Energy Differences in Nonequilibrium Work SQM/MM Switching Simulations
Source: J Phys Chem B. 2022 Apr 11;126(15):2798–811. doi: 10.1021/acs.jpcb.2c00696 (PMC9036525; doi:10.1021/acs.jpcb.2c00696)
Supplement: Supplementary file 1 — jp2c00696_si_001.pdf [file jp2c00696_si_001.pdf]

Supporting Information for Publication Optimizing the Calculation of Free Energy Differences in Nonequilibrium Work SQM/MM Switching Simulations

Andreas Schöller,[†,¶] Fiona Kearns,[‡] H. Lee Woodcock,[*,‡] and Stefan Boresch[*,†]

†*University of Vienna, Faculty of Chemistry, Department of Computational Biological Chemistry, Währingerstr. 17, A-1090 Vienna, Austria*

‡*Department of Chemistry, University of South Florida, 4202 E. Fowler Ave., CHE205, Tampa, FL 33620-5250, United States*

¶*University of Vienna, Vienna Doctoral School in Chemistry (DoSChem), Währingerstr. 42, A-1090 Vienna, Austria*

E-mail: hlw@usf.edu; stefan.boresch@univie.ac.at

List of Tables

S1 Additional details of model compounds S3

S2 Summary Statistics for Short Equilibrations S3

S3 Detailed results for N_{switch}=200 fs $N_{replicate}$=8000 . . . . S5

S4 Detailed results for N_{switch}=500 fs $N_{replicate}$=3200 . . . . S5

S5 Detailed results for N_{switch}=1000 fs $N_{replicate}$=1600 . . . . S6

S6 Detailed results for N_{switch}=2000 fs $N_{replicate}$=800 . . . . S6

List of Figures

S1 Changes in dihedral angle nomenclature of **7** S7

S2 Potential energy scan for **7** S8

S3 Initial $\delta\Delta A$ Results with larger scale S8

S4 Initial $\delta\Delta A$ Results including error bars S9

S5 Final $\delta\Delta A$ Results including error bars S9

S6 Quasi time series of compound **2** S10

S7 Quasi time series of compound **7** S10

S8 Results modified equilibration protocol S11

S9 Principal component analysis of compound **2** S12

S10 Principal component analysis of compound **7** S12

Tab. S1. ZINC ID,[1] ID used in Ref.[2] and in this manuscript, total number of atoms (N_{total}), the total number of heavy atoms (N_{heavy}) and free energy offset for each compound investigated. This offset has to be subtracted from the respective $\Delta A^{MM \rightarrow SQM}$ ($\Delta A^{SQM \rightarrow MM}$) listed in Tables S3–S6 below.

ZINC-ID	ID	N_{tot}/N_{heavy}	Offset (kcal/mol)
00077329	2	16/10	15000
00079729	3	18/13	17000
00086442	4	21/12	19000
00107550	7	21/11	16000
00133435	10	34/22	28000
00138607	11	36/20	29000
00140610	12	20/12	17000
00164361	13	23/14	20000
00167648	14	44/26	35000
00169358	15	26/16	22000
01867000	17	32/18	22000
06568023	21	30/18	25000

Tab. S2. MAD (Mean Absolute Deviation) and RMSE (Root Mean Square Error) of results obtained from forward switches compared to Reference result (CRO). These are the values resulting from the short equilibration protocols used initially; this table corresponds to Fig. 3 of the main manuscript.

	MAD [kcal/mol]	RMSE [kcal/mol]
NSWI200	0.80	1.79
NSWI500	0.56	1.36
NSWI1000	0.49	1.07
NSWI2000	0.20	0.36

Convergence criteria reported in Tables S3–S6: In line with earlier work,[2] we computed several convergence criteria for each free energy difference $\Delta A^{MM \rightarrow SQM}$. As described in the main manuscript, raw data for each ΔA were generated through eight independent series of calculations. We consider each set of raw data as a block and calculated ΔA_i for each of these blocks. Thus, we can compute a "hysteresis" Hyst(eresis) $= \Delta A - 1/8 \sum_{i=1}^{8} \Delta A_i$; cf. Ref. 3. Here, ΔA is the free energy computed using Jarzynski's equation from *all* available data, combining the work values of the eight series of calculations. Each ΔA_i is the free energy computed from the work values of one of the eight series of calculations.

Further, we report the one-sided Π values for the forward and backward free energy differences; see Ref. 4 for the definition of this quantity and the rationale behind it. Π values > 0.5 indicate that the W distribution is based on sufficient and unbiased sampling. We report two variants for Π, one evaluated with the initial guess for ΔA calculated by CRO (Π_{CRO}), the other by JAR (Π_{JAR}). Here (cf. main manuscript) CRO refers to Crook's equation,[5] and JAR to Jarzynski's equation.[6]

Tab. S3. Detailed results for N_{switch}=200 fs and $N_{replicate}$=8000 : $\Delta A_{gas}^{MM \rightarrow SQM}$ calculated with JAR (fw), JAR (bw), and CRO, as well as relevant convergence metrics, i.e., standard deviation $\sigma \Delta Ai$ for the 8 blocks, the average work $\overline{W}$, the standard deviation of W (σW), the one-sided Π_{CRO} and Π_{JAR} values, as well as percentage overlap for the distribution of work values between the JAR(fw) and JAR(bw) calculations. For compounds 2, 7, 10, 12 and 21 entries in italics are for the original protocol, entries in bold indicate the results obtained with longer equilibration after the random dihedral assignment.

	JAR(fw)							JAR(bw)							CRO		Overlap (%)
Paper-ID	ΔA	Hyst	σ ΔAi	$\overline{W}$	σW	Π_{CRO}	Π_{JAR}	ΔA	Hyst	σ ΔAi	$\overline{W}$	σW	Π_{CRO}	Π_{JAR}	ΔA	σ ΔA	W
2	*-258.08*	*0.28*	*0.59*	*-256.35*	*1.33*	*1.36*	*1.27*	*257.39*	*0.11*	*0.39*	*258.82*	*2.11*	*1.99*	*1.49*	*-257.44*	*0.02*	*26.37*
**2**	**-257.84**	**0.28**	**0.55**	**-256.02**	**1.26**	**0.55**	**1.20**	**257.17**	**0.92**	**1.78**	**259.91**	**2.76**	**2.13**	**0.64**	**-257.37**	**0.02**	**20.35**
3	-414.30	0.01	0.10	-412.75	1.50	2.22	1.39	413.90	0.02	0.15	415.05	1.00	1.32	1.72	-414.08	0.02	22.62
4	-256.52	0.00	0.03	-256.00	0.79	2.59	2.36	256.04	0.00	0.03	256.58	0.86	2.81	2.34	-256.28	0.01	58.07
7	*-994.14*	*0.39*	*0.75*	*-992.56*	*1.08*	*0.49*	*1.37*	*993.21*	*1.27*	*2.00*	*996.10*	*3.00*	*2.88*	*0.57*	*-993.57*	*0.02*	*26.04*
**7**	**-993.20**	**0.19**	**0.43**	**-992.05**	**0.96**	**1.59**	**1.71**	**992.59**	**0.03**	**0.21**	**993.65**	**1.51**	**2.61**	**1.79**	**-992.71**	**0.01**	**35.29**
10	*-338.40*	*0.28*	*0.59*	*-335.73*	*1.72*	*0.92*	*0.69*	*337.79*	*0.06*	*0.26*	*340.23*	*1.71*	*0.95*	*0.82*	*-337.97*	*0.03*	*11.04*
**10**	**-338.30**	**0.20**	**0.53**	**-335.67**	**1.67**	**0.88**	**0.71**	**337.91**	**0.06**	**0.26**	**340.22**	**1.70**	**0.96**	**0.89**	**-337.90**	**0.03**	**10.81**
11	-462.22	0.07	0.33	-459.19	3.09	1.73	0.49	461.95	0.00	0.12	463.65	1.64	0.47	1.29	-462.00	0.02	21.64
12	*-76.70*	*0.06*	*0.19*	*-75.51*	*1.06*	*1.73*	*1.68*	*75.78*	*0.03*	*0.12*	*77.12*	*1.38*	*2.40*	*1.56*	*-76.22*	*0.00*	*33.82*
**12**	**-76.68**	**0.04**	**0.22**	**-75.50**	**1.06**	**1.76**	**1.67**	**75.82**	**0.05**	**0.08**	**77.09**	**1.37**	**2.38**	**1.61**	**-76.21**	**0.02**	**34.37**
13	-567.91	0.00	0.04	-567.44	0.75	1.73	1.68	567.32	0.00	0.04	567.81	0.78	2.40	1.56	-567.62	0.01	68.17
14	-82.73	0.04	0.23	-81.35	1.20	1.41	1.53	82.36	0.01	0.10	83.96	1.48	1.51	1.36	-82.57	0.02	20.31
15	-407.80	0.00	0.06	-407.38	0.64	2.24	2.50	407.49	0.00	0.02	407.99	0.88	3.19	2.38	-407.64	0.01	52.92
17	-673.51	0.00	0.07	-672.91	0.81	2.31	2.26	673.21	0.00	0.05	673.85	0.90	2.54	2.22	-673.36	0.01	41.08
21	*-75.12*	*4.22*	*3.09*	*-68.27*	*1.61*	*0.92*	*-1.12*	*68.80*	*0.01*	*0.07*	*70.75*	*2.14*	*2.88*	*1.12*	*-69.08*	*0.02*	*24.14*
**21**	**-69.00**	**0.01**	**0.11**	**-68.00**	**0.95**	**0.94**	**1.84**	**68.78**	**0.01**	**0.08**	**70.74**	**2.16**	**2.48**	**1.12**	**-68.99**	**0.03**	**23.31**

Tab. S4. Detailed results for N_{switch}=500 fs $N_{replicate}$=3200

	JAR(fw)							JAR(bw)							CRO		Overlap (%)
Paper-ID	ΔA	Hyst	σ ΔAi	$\overline{W}$	σW	Π_{CRO}	Π_{JAR}	ΔA	Hyst	σ ΔAi	$\overline{W}$	σW	Π_{CRO}	Π_{JAR}	ΔA	σ ΔA	W
2	*-258.14*	*0.37*	*0.55*	*-256.97*	*0.83*	*1.33*	*1.46*	*257.43*	*0.02*	*0.20*	*258.36*	*1.71*	*2.75*	*1.68*	*-257.48*	*0.02*	*37.72*
**2**	**-257.38**	**0.04**	**0.21**	**-256.73**	**0.77**	**0.69**	**1.96**	**257.28**	**0.79**	**1.61**	**259.02**	**2.25**	**3.13**	**1.02**	**-257.37**	**0.02**	**33.93**
3	-414.23	0.01	0.10	-413.23	1.27	2.44	1.61	413.95	0.06	0.26	414.72	0.77	1.42	1.84	-414.11	0.03	31.41
4	-256.39	0.00	0.07	-256.12	0.54	2.59	2.49	256.19	0.00	0.03	256.45	0.59	2.83	2.51	-256.28	0.02	64.48
7	*-993.87*	*0.23*	*0.60*	*-992.91*	*0.83*	*0.46*	*1.64*	*993.28*	*1.28*	*2.02*	*995.68*	*2.79*	*3.35*	*0.60*	*-993.59*	*0.02*	*32.58*
**7**	**-992.79**	**0.01**	**0.12**	**-992.35**	**0.67**	**1.60**	**2.23**	**992.64**	**0.03**	**0.21**	**993.31**	**1.21**	**2.96**	**1.94**	**-992.72**	**0.02**	**46.86**
10	*-338.38*	*0.25*	*0.58*	*-336.83*	*1.20*	*1.21*	*1.16*	*337.88*	*0.01*	*0.10*	*339.27*	*1.47*	*1.66*	*1.28*	*-337.94*	*0.03*	*22.51*
**10**	**-337.92**	**0.03**	**0.20**	**-336.75**	**1.13**	**1.17**	**1.46**	**337.73**	**0.01**	**0.10**	**339.30**	**1.51**	**1.66**	**1.14**	**-337.88**	**0.03**	**21.59**
11	-462.11	0.06	0.19	-459.72	2.90	2.27	0.61	461.99	0.00	0.17	463.06	1.17	0.46	1.55	-461.99	0.03	28.04
12	*-76.37*	*0.00*	*0.05*	*-75.87*	*0.75*	*2.13*	*2.14*	*76.03*	*0.01*	*0.03*	*76.60*	*0.88*	*2.57*	*2.06*	*-76.20*	*0.02*	*50.00*
**12**	**-76.38**	**0.01**	**0.05**	**-75.87**	**0.74**	**2.11**	**2.13**	**76.04**	**0.01**	**0.06**	**76.60**	**0.88**	**2.58**	**2.07**	**-76.20**	**0.02**	**49.03**
13	-567.72	0.00	0.01	-567.52	0.49	2.13	2.14	567.48	0.00	0.02	567.70	0.51	2.57	2.06	-567.61	0.02	75.75
14	-82.65	0.00	0.06	-81.62	1.03	1.53	1.58	82.52	0.01	0.19	83.60	1.16	1.71	1.54	-82.58	0.03	22.59
15	-407.69	0.01	0.04	-407.48	0.48	2.42	2.58	407.57	0.00	0.03	407.82	0.59	2.98	2.54	-407.63	0.02	59.33
17	-673.41	0.00	0.03	-673.17	0.53	2.55	2.54	673.30	0.00	0.03	673.55	0.57	2.74	2.52	-673.36	0.02	58.35
21	*-73.64*	*0.88*	*1.01*	*-68.62*	*1.46*	*1.28*	*-0.67*	*68.91*	*0.00*	*0.03*	*70.06*	*1.54*	*3.66*	*1.48*	*-69.07*	*0.02*	*32.66*
**21**	**-69.05**	**0.01**	**0.12**	**-68.34**	**0.78**	**1.22**	**1.90**	**68.88**	**0.01**	**0.09**	**70.02**	**1.53**	**2.52**	**1.15**	**-68.98**	**0.02**	**30.68**

Tab. S5. Detailed results for N_{switch}=1000 fs $N_{replicate}$=1600

	JAR(fw)							JAR(bw)							CRO		Overlap (%)
Paper-ID	ΔA	Hyst	σ ΔAi	$\overline{W}$	σW	Π_{CRO}	Π_{JAR}	ΔA	Hyst	σ ΔAi	$\overline{W}$	σW	Π_{CRO}	Π_{JAR}	ΔA	σ ΔA	W
2	*-257.54*	*0.04*	*0.22*	*-257.12*	*0.61*	*1.20*	*2.07*	*257.46*	*0.12*	*0.39*	*258.20*	*1.57*	*3.06*	*1.68*	*-257.48*	*0.03*	*43.15*
**2**	**-257.49**	**0.08**	**0.32**	**-256.94**	**0.57**	**0.66**	**1.90**	**257.34**	**0.79**	**1.59**	**258.73**	**2.01**	**3.48**	**1.09**	**-257.39**	**0.04**	**39.58**
3	-414.18	0.00	0.05	-413.65	0.93	2.49	1.92	413.94	0.08	0.28	414.49	0.64	1.71	1.89	-414.13	0.03	42.57
4	-256.35	0.00	0.02	-256.21	0.41	2.55	2.55	256.25	0.00	0.02	256.40	0.45	2.82	2.54	-256.30	0.02	70.56
7	*-995.26*	*1.30*	*1.22*	*-993.03*	*0.74*	*0.41*	*0.52*	*993.28*	*1.26*	*1.95*	*995.47*	*2.69*	*3.44*	*0.54*	*-993.59*	*0.03*	*33.11*
**7**	**-992.84**	**0.03**	**0.21**	**-992.44**	**0.52**	**1.53**	**2.10**	**992.70**	**0.02**	**0.14**	**993.16**	**1.02**	**3.03**	**2.00**	**-992.71**	**0.03**	**51.53**
10	*-338.34*	*0.20*	*0.47*	*-337.35*	*0.89*	*1.35*	*1.42*	*337.87*	*0.01*	*0.08*	*338.74*	*1.24*	*2.19*	*1.54*	*-337.93*	*0.03*	*34.57*
**10**	**-337.91**	**0.03**	**0.18**	**-337.27**	**0.84**	**1.35**	**1.78**	**337.85**	**0.01**	**0.08**	**338.75**	**1.25**	**2.09**	**1.52**	**-337.90**	**0.03**	**33.74**
11	-462.14	0.06	0.28	-460.17	2.63	2.61	0.68	462.08	0.01	0.12	462.76	0.89	0.49	1.75	-462.01	0.04	34.43
12	*-76.31*	*0.00*	*0.03*	*-76.04*	*0.55*	*2.31*	*2.30*	*76.12*	*0.00*	*0.06*	*76.41*	*0.62*	*2.62*	*2.26*	*-76.21*	*0.03*	*62.42*
**12**	**-76.29**	**0.01**	**0.06**	**-76.02**	**0.54**	**2.28**	**2.31**	**76.14**	**0.01**	**0.07**	**76.44**	**0.62**	**2.56**	**2.25**	**-76.22**	**0.03**	**57.54**
13	-567.69	0.00	0.02	-567.58	0.37	2.31	2.30	567.55	0.00	0.04	567.66	0.37	2.62	2.26	-567.62	0.02	84.06
14	-82.50	0.04	0.23	-81.78	0.90	1.59	1.69	82.65	0.01	0.11	83.34	0.93	1.66	1.72	-82.55	0.03	25.32
15	-407.66	0.00	0.03	-407.52	0.38	2.49	2.58	407.59	0.00	0.04	407.73	0.43	2.84	2.56	-407.62	0.02	66.86
17	-673.39	0.00	0.03	-673.27	0.38	2.59	2.61	673.34	0.00	0.03	673.48	0.41	2.73	2.58	-673.37	0.02	67.30
21	*-72.31*	*2.14*	*1.81*	*-68.76*	*1.14*	*1.65*	*-0.20*	*68.93*	*0.00*	*0.06*	*69.63*	*1.14*	*3.74*	*1.72*	*-69.04*	*0.03*	*40.53*
**21**	**-69.19**	**0.09**	**0.31**	**-68.57**	**0.65**	**1.46**	**1.80**	**68.98**	**0.01**	**0.07**	**69.66**	**1.09**	**2.44**	**1.74**	**-69.01**	**0.03**	**37.09**

Tab. S6. Detailed results for N_{switch}=2000 fs $N_{replicate}$=800

	JAR(fw)							JAR(bw)							CRO		Overlap (%)
Paper-ID	ΔA	Hyst	σ ΔAi	$\overline{W}$	σW	Π_{CRO}	Π_{JAR}	ΔA	Hyst	σ ΔAi	$\overline{W}$	σW	Π_{CRO}	Π_{JAR}	ΔA	σ ΔA	W
2	*-258.05*	*0.36*	*0.69*	*-257.21*	*0.55*	*1.05*	*1.37*	*257.48*	*0.11*	*0.37*	*258.11*	*1.47*	*3.18*	*1.59*	*-257.48*	*0.04*	*46.51*
**2**	**-257.53**	**0.16**	**0.43**	**-257.03**	**0.42**	**0.57**	**1.76**	**257.36**	**0.65**	**1.40**	**258.58**	**1.90**	**3.61**	**1.03**	**-257.34**	**0.04**	**39.58**
3	-414.12	0.00	0.06	-413.87	0.59	2.38	2.14	414.07	0.02	0.17	414.33	0.48	1.98	2.11	-414.12	0.04	48.74
4	-256.32	0.00	0.04	-256.25	0.30	2.42	2.54	256.26	0.00	0.02	256.34	0.34	2.91	2.54	-256.29	0.03	79.22
7	*-993.91*	*0.34*	*0.71*	*-993.13*	*0.66*	*0.33*	*1.44*	*993.28*	*1.24*	*1.91*	*995.37*	*2.65*	*3.42*	*0.41*	*-993.61*	*0.04*	*33.62*
**7**	**-992.78**	**0.04**	**0.22**	**-992.51**	**0.39**	**1.33**	**2.11**	**992.71**	**0.02**	**0.17**	**993.11**	**0.98**	**3.19**	**1.88**	**-992.71**	**0.03**	**56.09**
10	*-338.34*	*0.19*	*0.48*	*-337.60*	*0.67*	*1.34*	*1.48*	*337.91*	*0.01*	*0.12*	*338.49*	*1.05*	*2.50*	*1.66*	*-337.94*	*0.04*	*43.62*
**10**	**-337.95**	**0.05**	**0.25**	**-337.51**	**0.61**	**1.36**	**1.83**	**337.89**	**0.01**	**0.12**	**338.46**	**1.06**	**2.37**	**1.67**	**-337.89**	**0.04**	**44.02**
11	-462.02	0.06	0.31	-460.62	2.29	2.82	0.88	461.18	0.55	0.72	462.54	0.72	0.54	0.92	-462.01	0.05	37.35
12	*-76.26*	*0.00*	*0.03*	*-76.13*	*0.38*	*2.42*	*2.40*	*76.18*	*0.00*	*0.03*	*76.32*	*0.42*	*2.56*	*2.36*	*-76.22*	*0.03*	*69.52*
**12**	**-76.24**	**0.01**	**0.04**	**-76.11**	**0.39**	**2.34**	**2.39**	**76.19**	**0.01**	**0.05**	**76.35**	**0.44**	**2.50**	**2.33**	**-76.22**	**0.03**	**65.29**
13	-567.66	0.00	0.02	-567.60	0.27	2.42	2.40	567.60	0.00	0.02	567.65	0.27	2.56	2.36	-567.62	0.03	86.77
14	-82.52	0.02	0.15	-81.94	0.80	1.65	1.66	82.62	0.01	0.12	83.14	0.77	1.63	1.73	-82.54	0.04	29.65
15	-407.65	0.00	0.05	-407.58	0.29	2.51	2.56	407.61	0.00	0.03	407.69	0.31	2.73	2.54	-407.63	0.03	75.41
17	-673.38	0.00	0.03	-673.31	0.28	2.58	2.58	673.35	0.00	0.04	673.42	0.30	2.66	2.56	-673.37	0.03	73.05
21	*-70.02*	*0.61*	*0.76*	*-68.79*	*0.74*	*2.08*	*1.02*	*68.99*	*0.01*	*0.10*	*69.37*	*0.79*	*3.61*	*1.93*	*-69.01*	*0.04*	*46.79*
**21**	**-69.00**	**0.01**	**0.09**	**-68.73**	**0.53**	**1.69**	**2.09**	**68.96**	**0.01**	**0.09**	**69.38**	**0.82**	**2.43**	**1.86**	**-69.01**	**0.04**	**47.98**

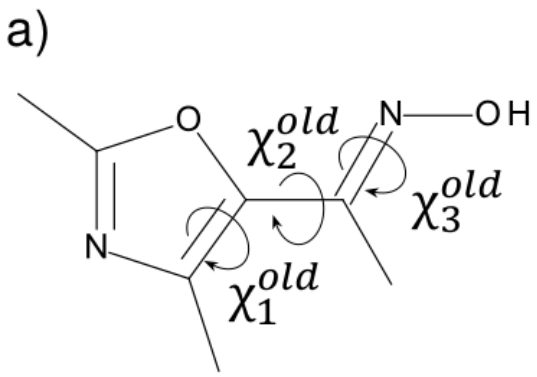

Fig. S1. Changes in dihedral angle nomenclature for compound **7**; a) dihedral labels used in Ref.[2]; b) dihedral labels used in this work.

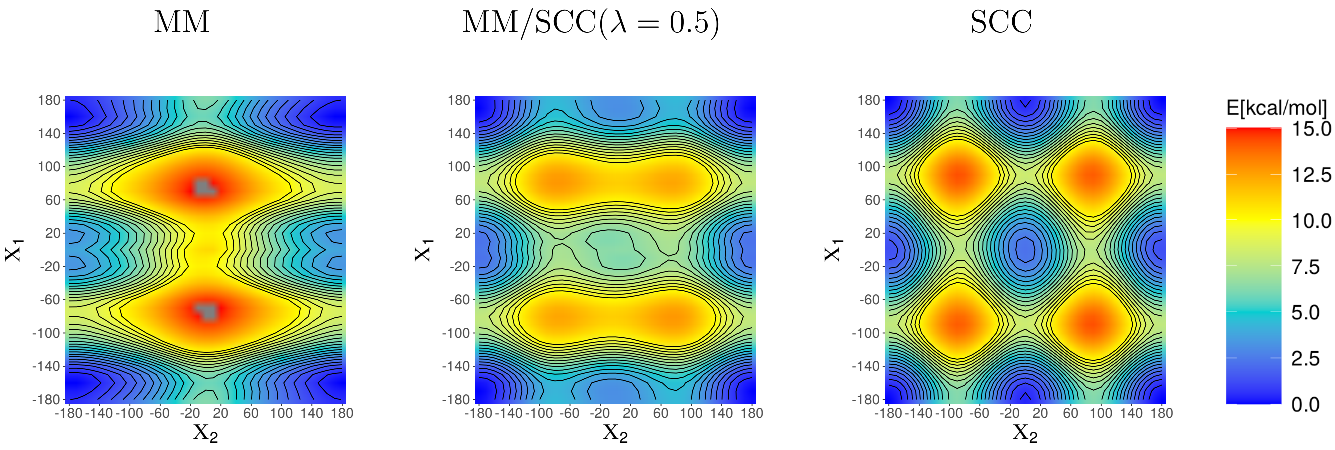

Fig. S2. Potential energy scan as a function of χ_1 and χ_2 (cf. Fig. 1 of the main manuscript and Fig. S1 above) for compound **7**. Left: MM; middle MM/3ob at $\lambda = 0.5$; right: SQM (=3ob). All energies are in kcal/mol, the contour lines are plotted in 0.5 kcal/mol increments from 0 to 10 kcal/mol. Energies were rescaled so that the minimum energy is at 0 kcal/mol in all three plots.

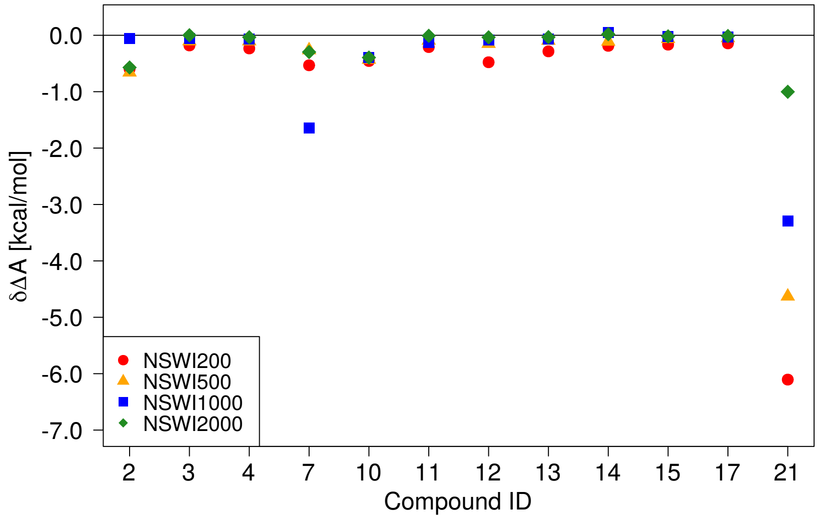

Fig. S3. $\delta\Delta A$ for the results of the forward switching simulations (MM $\rightarrow$ SQM) using the original equilibration protocol as a function of switching length. Here all values, including those that were off-scale in Fig. 3 of the manuscript, are shown.

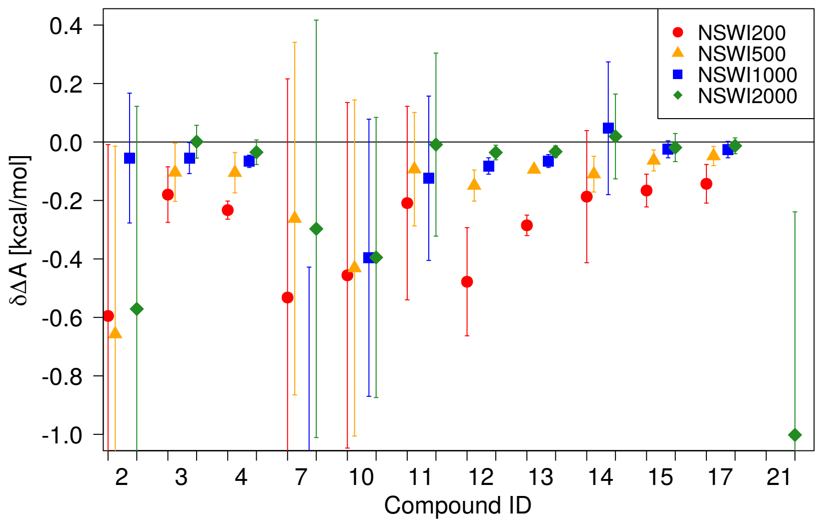

Fig. S4. $\delta\Delta A$ as shown in Fig. 3 of the main manuscript (original equilibration protocol) with error bars, based on $\sigma_{\Delta A_i}$ from Tables S4-S7.

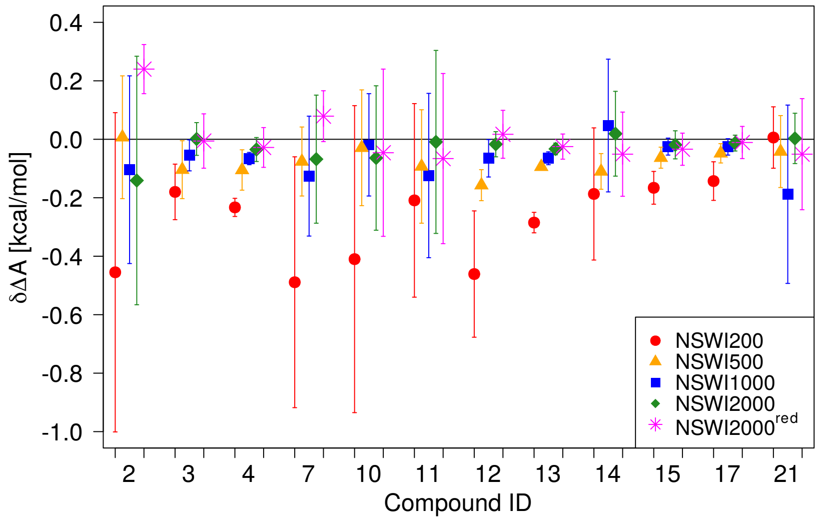

Fig. S5. $\delta\Delta A$ as shown in Fig. 7 of the main manuscript (longer equilibration for **2**, **7**, **10**, **12** and **21**) with error bars, based on $\sigma_{\Delta A_i}$ from Tables S4-S7.

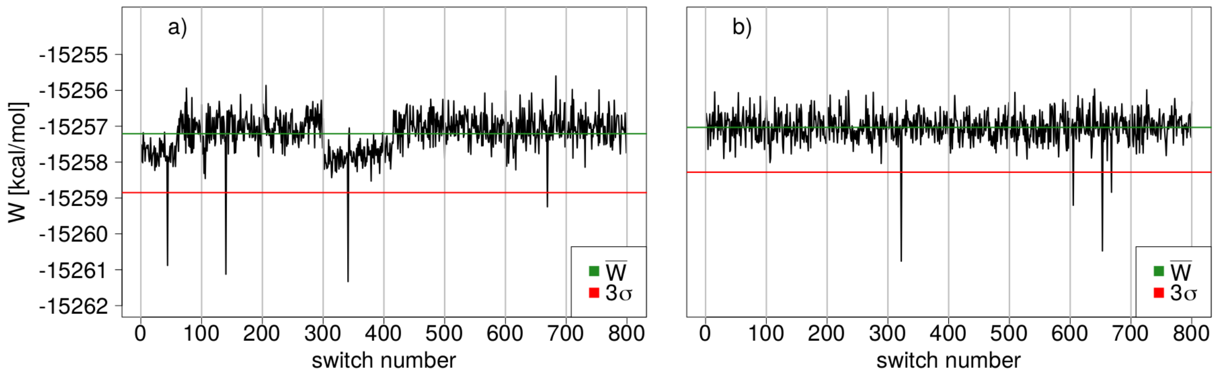

Fig. S6. Quasi time series of compound **2** for a) the original protocol and for b) the modified equilibration protocol.

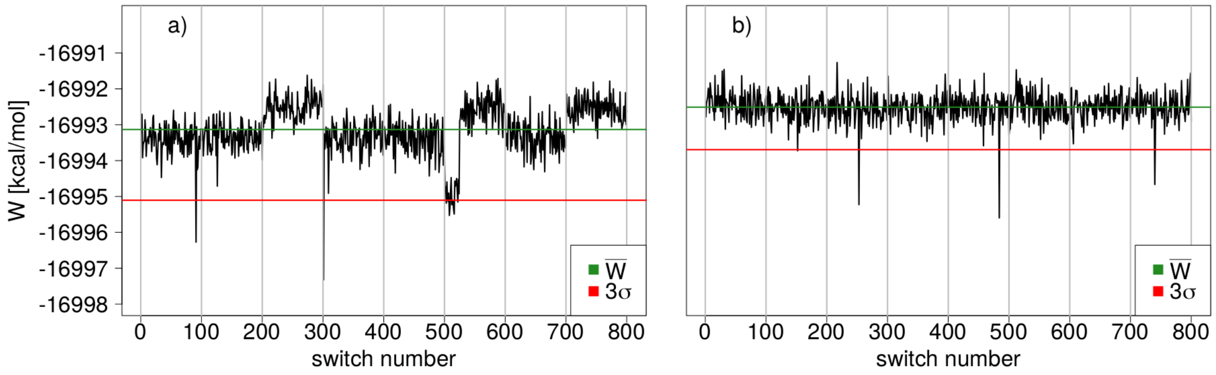

Fig. S7. Quasi time series of compound **7** for a) the original protocol and for b) the modified equilibration protocol.

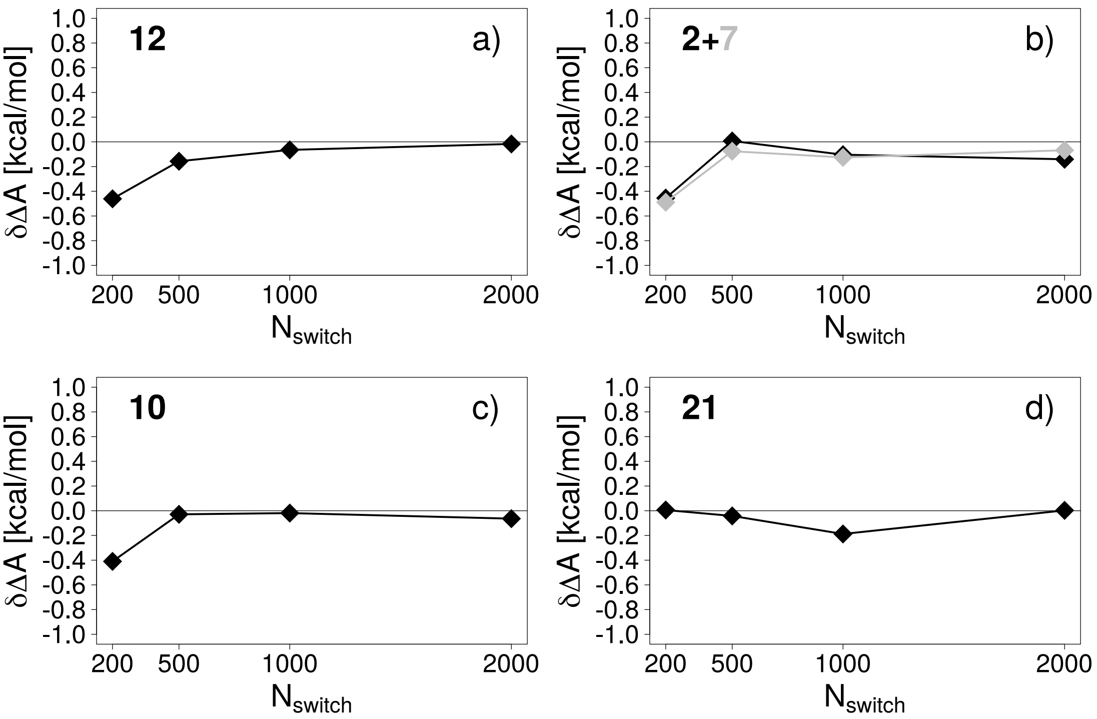

Fig. S8. Detailed dependence of $\delta\Delta A$ on switching length for a) compounds **12** b) **2** and **7**, c)**10** and d) **21** with extended pre-production equilibration time. This is the counterpart to Fig. 4 of the main manuscript.

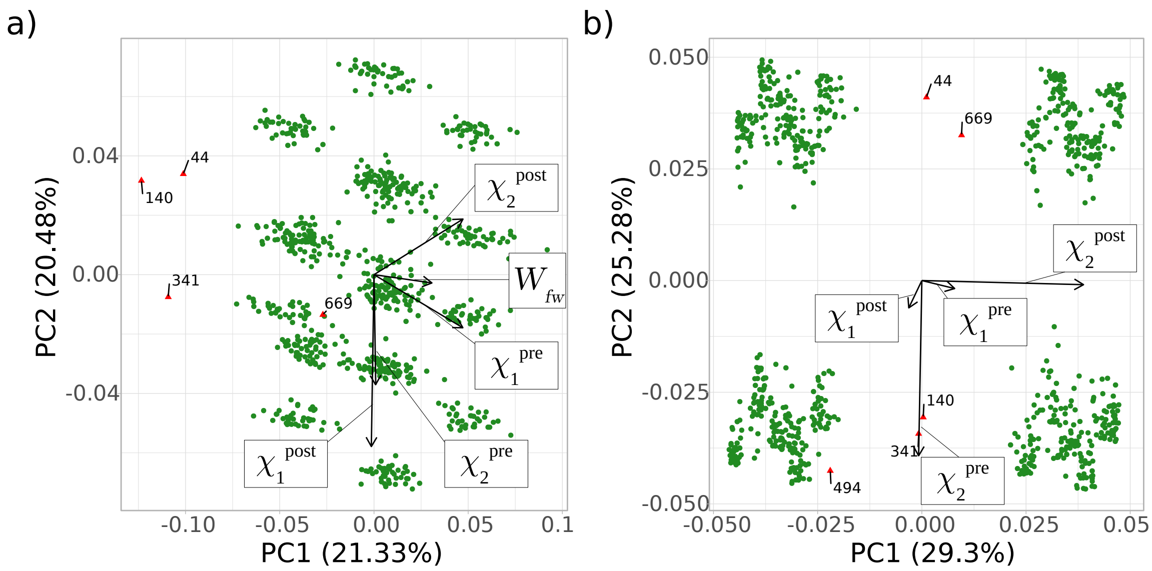

Fig. S9. PCA of compound **2** plotted as PC1 vs. PC2 for the original protocol. a) Scaled version; b) unscaled version. Outliers are marked according to predefined criteria described in Sect. 3.3 of the main manuscript; red triangles (rather than green dots) indicate deviations a) lower than $\bar{W} - 3\sigma$, and b) $|\bar{\chi}_2^{post}| - 3\sigma$.

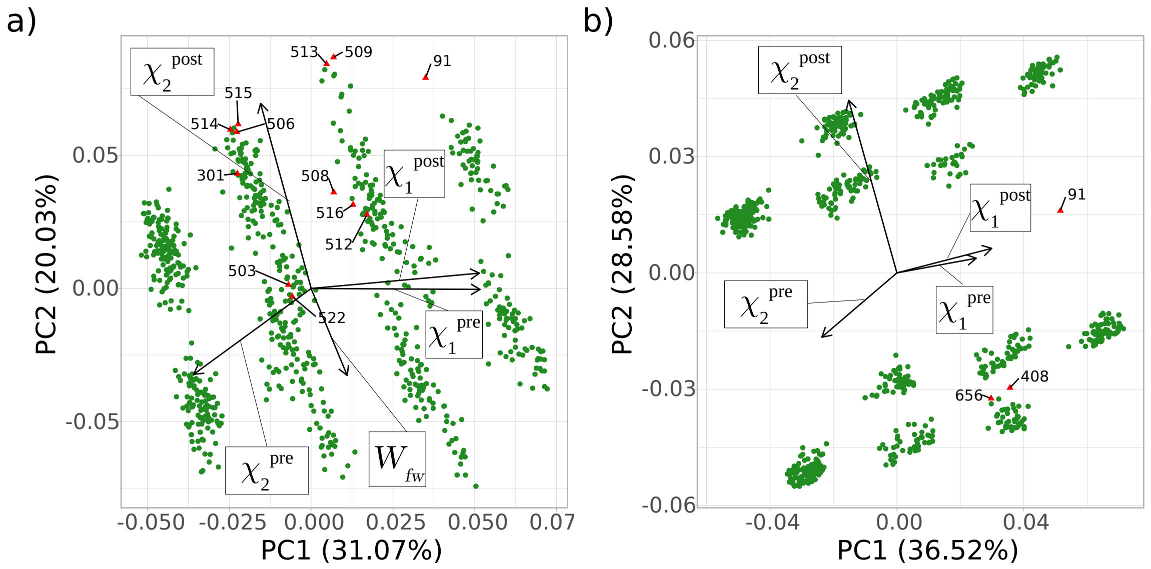

Fig. S10. PCA of compound **7** plotted as PC1 vs. PC2 for the original protocol. a) Scaled version; b) unscaled version. Outliers are marked according to predefined criteria described in Sect. 3.3 of the main manuscript; red triangles (rather than green dots) indicate deviations a) lower than $\bar{W} - 3\sigma$, and b) $|\bar{\chi}_2^{post}| - 3\sigma$.

References

(1) Irwin, J. J.; Sterling, T.; Mysinger, M. M.; Bolstad, E. S.; Coleman, R. G. ZINC: A Free Tool to Discover Chemistry for Biology. *J. Chem. Inf. Model.* **2012**, *52*, 1757–1768, PMID: 22587354.

(2) Kearns, F. L.; Warrensford, L.; Boresch, S.; Woodcock, H. L. The Good, the Bad, and the Ugly: "HiPen", a New Dataset for Validating (S)QM/MM Free Energy Simulations. *Molecules* **2019**, *24*, 681.

(3) Wood, R. H.; Muhlbauer, W. C. F.; Thompson, P. T. Systematic errors in free energy perturbation calculations due to a finite sample of configuration space: sample-size hysteresis. *J. Phys. Chem.* **1991**, *95*, 6670–6675.

(4) Wu, D.; Kofke, D. A. Model for small-sample bias of free-energy calculations applied to Gaussian-distributed nonequilibrium work measurements. *J. Chem. Phys.* **2004**, *121*, 8742–8747.

(5) Crooks, G. E. Path-ensemble averages in systems driven far from equilibrium. *Phys. Rev. E* **2000**, *61*, 2361.

(6) Jarzynski, C. Nonequilibrium equality for free energy differences. *Phys. Rev. Lett.* **1997**, *78*, 2690.
